# Three-in-One: Dye-Volatile Cocrystals Exhibiting Intensity-Dependent Photochromic, Photomechanical, and Photocarving Response

**DOI:** 10.1021/jacs.3c07060

**Published:** 2023-11-04

**Authors:** Tristan H. Borchers, Filip Topić, Mihails Arhangelskis, Jogirdas Vainauskas, Hatem M. Titi, Oleksandr S. Bushuyev, Christopher J. Barrett, Tomislav Friščić

**Affiliations:** † Department of Chemistry, McGill University, Montreal H3A 0B8, Canada; ‡ School of Chemistry, University of Birmingham, Birmingham B15 2TT, United Kingdom; § Faculty of Chemistry, University of Warsaw, Warsaw 02-093, Poland

## Abstract

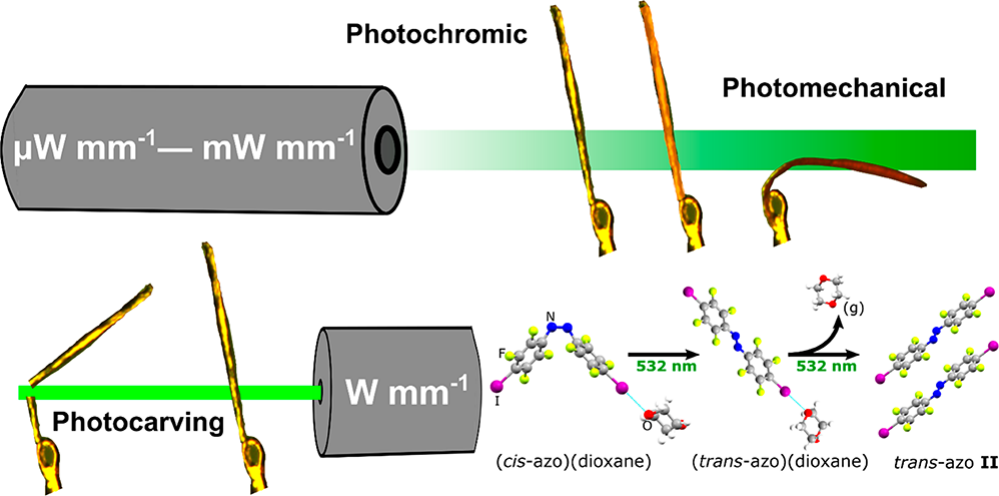

Cocrystallization of a *cis*-azobenzene dye with volatile molecules, such as pyrazine and dioxane, leads to materials that exhibit at least three different light-intensity-dependent responses upon irradiation with low-power visible light. The halogen-bond-driven assembly of the dye *cis*-(*p*-iodoperfluorophenyl)azobenzene with volatile halogen bond acceptors produces cocrystals whose light-induced behavior varies significantly depending on the intensity of the light applied. Low-intensity (<1 mW·cm^–2^) light irradiation leads to a color change associated with low levels of *cis* → *trans* isomerization. Irradiation at higher intensities (150 mW·mm^–2^) produces photomechanical bending, caused by more extensive isomerization of the dye. At still higher irradiation intensities (2.25 W·mm^–2^) the cocrystals undergo cold photocarving; i.e., they can be cut and written on with micrometer precision using laser light without a major thermal effect. Real-time Raman spectroscopy shows that this novel photochemical behavior differs from what would be expected from thermal energy input alone. Overall, this work introduces a rational blueprint, based on supramolecular chemistry in the solid state, for new types of crystalline light-responsive materials, which not only respond to being exposed to light but also change their response based on the light intensity.

## Introduction

Modulating the optical or photoresponsive properties of solid-state materials by light, i.e., inducing changes in shape,^1,2^ reversible or irreversible photomechanical motion,^3−9^ or optical properties,^10^ is of great interest for applications including molecular robotics,^11^ nanoactuators,^12^ and light harvesting.^13^ Control over optical properties of crystalline materials has attracted attention in the context of organic semiconductors,^14^ organic light-emitting diodes (OLEDs),^15^ elastic materials,^16^ or waveguides.^17,18^ Photoresponsive behavior in organic solids has been accomplished through a wide range of transformations, including diverse isomerization,^19,20^ polymerization,^15,16^ and dimerization reactions.^21−26^ The formation of multicomponent crystals (cocrystals) is a powerful crystal engineering and supramolecular strategy to tune the solid-state environment of an organic molecule, enabling the optimization of solid-state properties or even the development of materials with entirely new properties.^27,28^ Notable applications of cocrystal formation include the development of pharmaceutical solid forms,^29,30^ the design of mechano-, photo-, or thermoresponsive materials,^31,32^ organic semiconducting solids, optical materials, and more.^33^ Cocrystallization was also found to provide access to complex solid-state behavior, e.g., materials exhibiting different types of response depending on the choice of stimulus.^34^

The use of azobenzene-based (**azo**) dyes as cocrystal components was recently shown to enable access to materials with unique and controllable photomechanical or enhanced optical behavior, including crystal shaping, as well as dichroism and/or pleochroism by design.^35,36^ We have recently shown that cocrystallization of a *trans*-azobenzene (*trans*-**azo**) dye with a volatile cocrystal former enables the design of a new class of organic solids: dye-volatile cocrystals which can undergo “cold photocarving” (CPC), i.e., micrometer-precision cutting, shaping, or embossing upon irradiation with low-energy visible light, through an optical and not a thermal mechanism.^37^ While conventional machining and photolithography techniques have previously been used to cut or introduce micrometer-size engravings into polymers,^38^ polymeric resins,^39^ cocrystal thin films,^36^ photonic crystals,^40^ hyperbolic metamaterials,^41^ and nanostructures,^42^ such thermal processes typically require laser powers on the order of kW per mm^2^ to GW per mm^2^ or, as in the case of focused ion beam milling, the use of other types of high-energy beams.^43−45^ In contrast, the CPC behavior enabled through the dye-volatile cocrystal design does not involve high-temperature disruption of covalent bonds but instead gentle, low-temperature, and localized cleavage of noncovalent interactions, such as halogen bonds (XB).^46^ Notably, XBs were shown to be relevant for altering the photoresponsive behavior in liquid crystals and crystalline solids.^47^ As a result, CPC can achieve micrometer-precision surface and volume effects, using laser beams with only a small fraction of the intensity compared to those used for traditional machining and photolithography techniques.^38^

**Figure 1:**
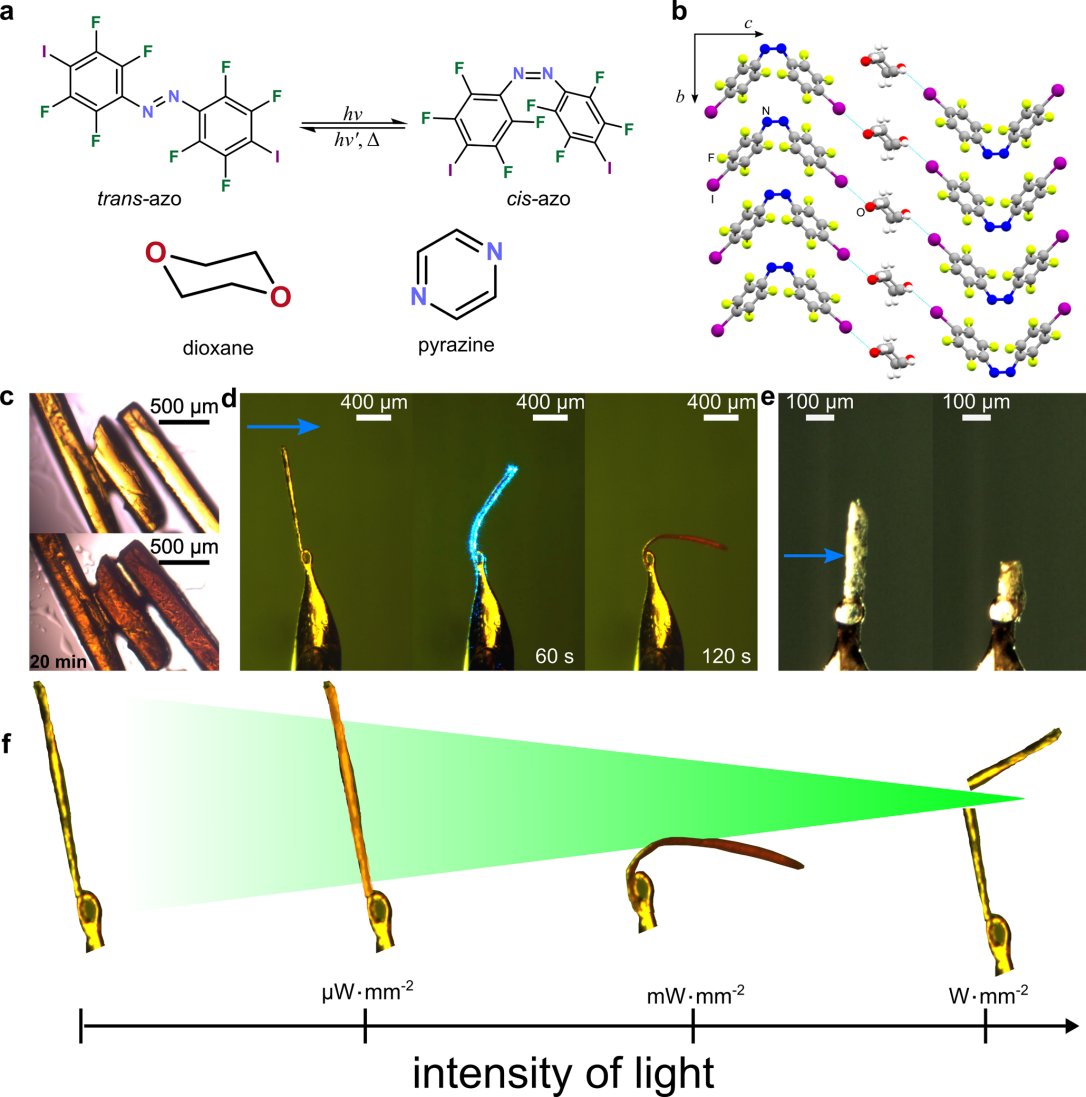
Illustration of three-way responsive *cis*-azobenzene cocrystals. (a) Schematic representation of **azo** isomerization and the molecular structures of the XB donor (*cis*-**azo**) and acceptor (dioxane, pyrazine) molecules used in this work. (b) Fragment of the crystal structure of the (*cis*-**azo**)(dioxane) cocrystal, viewed along the [100] crystallographic direction. (c) Images illustrating the optical color change occurring when (*cis*-**azo**)(dioxane) cocrystals are irradiated with dispersed, low-power 532 nm LED light. (d) Images illustrating the observed photomechanical bending of the (*cis*-**azo**)(dioxane) cocrystal upon irradiation by 488 nm light of power density 150 mW·mm^–2^ for different lengths of time. (e) Images illustrating the cold photocarving of a single (*cis*-**azo**)(dioxane) cocrystal upon exposure to a laser beam of 532 nm wavelength and light intensity of 2.25 W·mm^–2^. (f) Schematic illustration of the multiresponsive behavior of the (*cis*-**azo**)(dioxane) and (*cis*-**azo**)(pyrazine) cocrystals upon irradiation with light of different intensities, controlled through modification of the beam diameter. In (d) and (e), the direction of the incident beam is indicated by the blue arrow.

Here we report that the use of a *cis*-azobenzene dye (*cis*-**azo**) as a component of the dye-volatile cocrystal design provides access to photoresponsive molecular crystals that can undergo at least three distinct types of photoinduced response upon visible light irradiation. Combining a fluorinated *cis*-**azo** XB donor with volatile XB acceptors dioxane or pyrazine produces cocrystals (*cis*-**azo**)(dioxane) and (*cis*-**azo**)(pyrazine) (Figure 1a) which selectively, and depending on the irradiation light intensity, undergo optical color change, photomechanical bending, or photocarving. Each of these optical responses, which result from using the photoswitchable *cis*-**azo** unit as a cocrystal component, can be achieved in the same crystal by careful control of laser irradiation parameters, including power and beam diameter.

## Results and Discussion

Yellow needle-shaped single crystals of (*cis*-**azo**)(dioxane) were obtained by slow evaporation of a solution of *cis*-**azo** in dioxane. Structural analysis by single crystal X-ray diffraction revealed that the structure consists of zigzag chains held together by I···O halogen bonds (Figure 1b, also Supplementary Figure 2). The I···O distance of 2.854(2) Å (corresponding to the RXB parameter;^48^ i.e., the ratio of the I···O distance to the sum of the van der Waals radii^49^ of O and I atoms of 0.815 was found to be notably shorter than for the analogous (*trans*-**azo**)(dioxane) cocrystal (*d*_I···O_ = 2.981(3) Å, RXB = 0.852).^37^ The photoresponsive behavior of (*cis*-**azo**)(dioxane) crystals was studied under irradiation with either blue or green light, using wavelength 488, 515, or 532 nm, in which *cis*-**azo** absorbs (Supplementary Figure 3). Irradiation at the lowest light intensity was achieved using a 532 nm dispersed light-emitting diode (LED) of approximately 37 mW cm^–2^ output power at the source, placed 10 cm away from the crystal. Experiments at higher light intensities were performed using an in-house laser system (Supplementary Figure 4), which included one or two separate laser beams. In a typical experiment, the laser beam was passed through a 50:50 beam splitter, with the transmitted beam passing through a series of optical elements to increase and collimate the size of the beam prior to reaching the sample. The reflected beam, on the other hand, was passed through a different series of optical elements, including a neutral density filter and a convex lens, which reduced the diameter and thus increased the intensity of the beam, reaching the sample orthogonal to the transmitted beam. The use of this system enabled simple, shutter-controlled switching between a laser beam of lower light intensity with a larger diameter (the transmitted beam) and a beam of higher light intensity with a lower diameter (the reflected beam). The beam diameters were determined using the “knife edge” technique to be 78 μm and 2 mm for the reflected and transmitted beams, respectively (Supplementary Figures 5 and 6).

The single crystals of (*cis*-**azo**)(dioxane) were found to exhibit three fundamentally different types of photoresponse upon irradiation, dependent on the light intensity employed, unlike the *trans*-**azo** cocrystals previously studied which only undergo a single photoresponse.^37^ The type of photoresponse was not noticeably dependent on the wavelength of incident irradiation (488, 515, or 532 nm), as long as either green or blue light was used, i.e. light with wavelengths that can excite well within the n−π* absorption band of the **azo** component (Supplementary Figure 3). Specifically, irradiation at low power intensities (below 1 mW cm^–2^) generated using the LED light source led only to a change in color (Figure 1c) of the single crystal, which was assignable to the isomerization of *cis*- to *trans*-**azo**. The extent of isomerization was estimated by irradiating a single crystal of (*cis*-**azo**)(dioxane) using the 532 nm LED source for 20 min, followed by dissolution in CDCl_3_ and ^19^F nuclear magnetic resonance spectroscopy (NMR) analysis. After overnight data collection, integration of the ^19^F NMR spectrum revealed the presence of *cis-* and *trans*-**azo** in a 92:8 stoichiometric ratio (Supplementary Figure 7). Analogous analysis of a nonirradiated (*cis*-**azo**)(dioxane) crystal from the same batch revealed no signal of the *trans*-isomer (Supplementary Figure 8), indicating that the color change upon LED irradiation is associated with approximately 8% extent of *cis* → *trans* isomerization.

**Figure 2:**
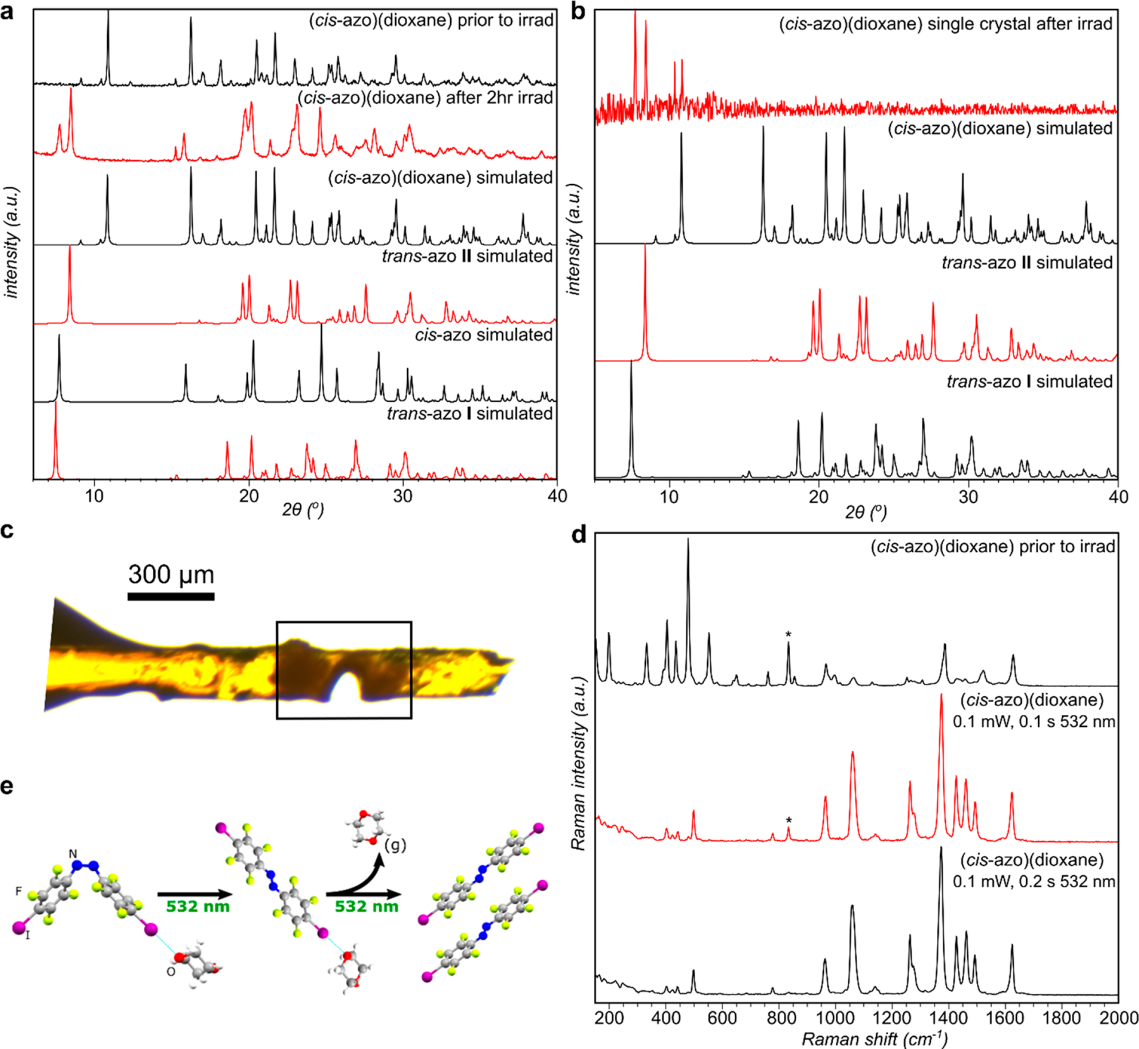
Photoresponse of (*cis*-**azo**)(dioxane) cocrystal studied by various methods. (a) PXRD analysis of bulk (*cis*-**azo**)(dioxane) sample before and after 2 h irradiation using a 532 nm, 37 mW cm^–2^ LED source. (b) PXRD pattern collected from the section highlighted in (c) of an irradiated single crystal of (*cis*-**azo**)(dioxane). (c) Optical image of a single crystal of (*cis*-**azo**)(dioxane) after cold photocarving using a 532 nm laser at an intensity of 2.25 W mm^–2^, with the rectangular box indicating the area over which PXRD analysis in panel b was performed. (d) Raman spectra from *in situ* analysis of a single crystal of (*cis*-**azo**)(dioxane) being irradiated with a 532 nm laser at a 0.1 mW power, illustrating first the replacement of *cis*-**azo** bands with those of *trans*-**azo** at 0.1 s of irradiation, followed by the disappearance of dioxane signal highlighted by “∗” after 0.2 s irradiation. (e) Schematic illustration of the phototransformation of (*cis*-**azo**)(dioxane) based on *in situ* Raman spectroscopy results shown in panel d.

The photochromic response at low light intensity was also evaluated on bulk powder of (*cis*-**azo**)(dioxane) by irradiation with a 532 nm LED source (37 mW cm^–2^). Powder X-ray diffraction (PXRD) analysis after 20 min irradiation showed the complete disappearance of Bragg reflections of (*cis*-**azo**)(dioxane) and the appearance of new reflections corresponding to the two previously reported polymorphs, *trans*-**azo I** and **II**, along with a small amount of solid *cis*-**azo** (Figure 2a).

Irradiation at a higher light intensity of 150 mW mm^–2^, achieved by switching to a laser light source with a beam diameter of 2 mm, produced not only a change in crystal color but also a strong photomechanical effect (Figure 1d, also Supporting Information Video 1 shown at 4× speed): the irradiated single crystals were observed to bend rapidly and significantly (within seconds) in a direction perpendicular to the long needle axis, corresponding to the (001) lattice plane (Supplementary Figure 9). The initial bending motion was toward the incident beam, followed by significant deflection in the opposite direction.^50^ The resulting curve-shaped crystal was orange-red in color, consistent with bending also being due to photoinduced *cis* → *trans* isomerization, specifically the rapid buildup of *trans*-**azo** on the irradiated crystal surface.^19^ Solution ^19^F NMR spectroscopy analysis of a (*cis*-**azo**)(dioxane) crystal that was irradiated for 1 min resulting in irreversible bending of ∼15° revealed a *cis*- to *trans*-isomers of 79:21. Compared to ∼8% conversion observed for the photochromic effect observed after 2 h irradiation with low intensity light, this indicates that the bending is associated with a faster buildup of the *trans*-isomer (Supplementary Figure 8). To better understand the relationship between the degrees of bending and the amount of isomerization,^51^ we also conducted photomechanical bending of two separate (*cis*-**azo**)(dioxane) cocrystals. After bending to approximately 20° and 50°, each cocrystal was analyzed by solution ^19^F NMR spectroscopy (Supplementary Figure 10). Although the resulting data are noisy due to instrumental limitation, there was a clear increase in the content of the *trans-*isomer of ∼33% and ∼60%, respectively.

Finally, switching to a laser beam of 78 μm diameter and light intensity of 2.25 W mm^–2^ resulted in cold photocarving (Figure 1e, also Supporting Information Video 2), evident by clean cutting of the crystal. In contrast to irradiation at lower power densities, which led to a change in color throughout the crystal, in this case, the color change from yellow to red was only observed on the irradiated spot. This observation suggests that only the directly irradiated part of the crystal undergoes isomerization. Analysis by PXRD (Figure 2b,c) of a single crystal of (*cis*-**azo**)(dioxane) that was cut using a higher light intensity (78 μm beam diameter) 532 nm laser beam, and then placed on a zero-background silicon sample holder, demonstrated the presence of *trans*-**azo** polymorphs **I** and **II** along with residual (*cis*-**azo**)(dioxane).

Consequently, single crystals of (*cis*-**azo**)(dioxane) were found to undergo three distinct types of photoresponse (Figure 1f): photochemical color change, photomechanical bending, and cold photocarving, each achievable through manipulating the intensity of the incident light. Moreover, eliciting a photoresponse at a lower light intensity did not prevent the cocrystal from also exhibiting a different response upon subsequent irradiation at a higher light intensity. For example, while irradiation of a crystal with a beam of 2 mm diameter led to photomechanical bending, subsequent irradiation of the same bent crystal with a beam of 78 μm diameter was still found to lead to photocarving. Such behavior provides a unique and, to the best of our knowledge, not yet reported opportunity to shape molecular crystals by carefully bending and cutting them using visible light.

### Raman Spectroscopy Analysis of Photoresponse

The photoresponsive behavior of a single crystal of (*cis*-**azo**)(dioxane) was then investigated *in situ* by Raman spectroscopy (Figure 2d, Supplementary Figure 11). Both the isomerization and dioxane loss from the crystal were probed on a confocal Raman microscope using a sequence of two rapid pulses (0.1 s each) of laser light of 532 nm wavelength and 0.1 mW power. After each pulse, the Raman spectrum of the irradiated spot on the crystal was immediately recorded using a 785 nm red laser, previously verified not to elicit a photoresponse. After the first 532 nm pulse, the Raman signals associated with the *cis*-**azo** component disappeared completely, concomitant with the appearance of new Raman bands associated with *trans*-**azo**. Importantly, the characteristic ν(C–O–C) Raman band for dioxane at 830 cm^–1^ remained, suggesting that irradiation induced a rapid transformation of (*cis*-**azo**)(dioxane) to the corresponding (*trans*-**azo**)(dioxane) cocrystal. Raman spectroscopy analysis of the irradiated spot following the second 532 nm laser pulse then revealed the complete disappearance of the ν(C–O–C) Raman band, with the overall spectrum exhibiting only the Raman shifts of pure *trans*-**azo**. This band disappearance suggests the complete loss of dioxane, consistent with the results of the PXRD analyses. Overall, the real-time Raman spectroscopy (Figure 2d) analyses on the single crystal indicate that the laser light-induced desolvation of (*cis*-**azo**)(dioxane) proceeds through a *cis* → *trans* photoisomerization, giving rise first to a transient appearance of (*trans*-**azo**)(dioxane), which subsequently converts to pure solid *trans*-**azo** (Figure 2e). Although the short-lived (*trans*-**azo**)(dioxane) phase was not observed by PXRD, such behavior is consistent with a previous *in situ* investigation of the irradiation of a *cis*-**azo** cocrystal with a nonvolatile coformer, which revealed a topotactic transformation to a corresponding *trans*-**azo** cocrystal.^35^

### Micrometer-Precision Photocarving

**Figure 3:**
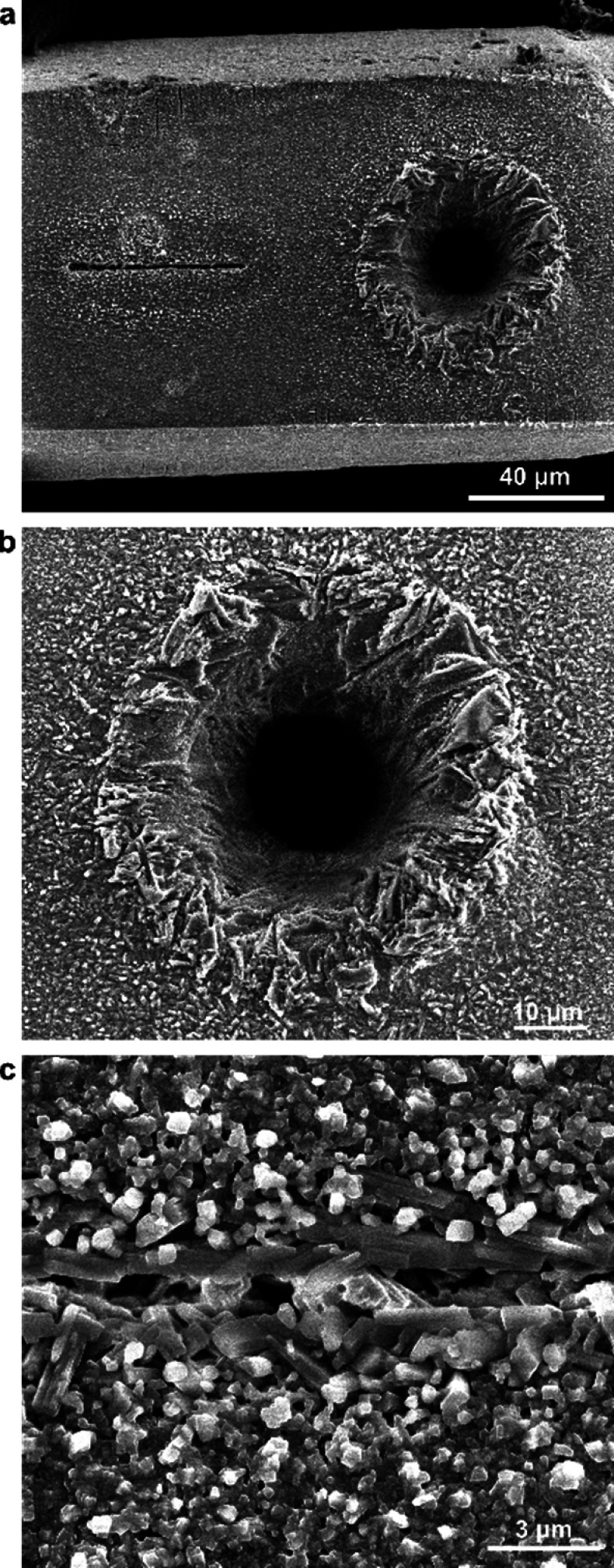
Selected SEM images of the surface of a (*cis*-**azo**)(dioxane) cocrystal after photocarving using a 532 nm beam in the confocal laser setup. (a) Line and hole made by using a laser beam of 1 and 5 mW power, respectively. (b) Close-up image of the hole, obtained by irradiating the crystal for 5 s with a 5 mW beam and (c) a linear cut created by dragging a 1 mW beam across the surface.

Next, we explored whether cutting and engraving of (*cis*-**azo**)(dioxane) cocrystal could proceed with the same micrometer-level precision that was recently reported for (*trans*-**azo**)(dioxane).^37^ Single crystals of (*cis*-**azo**)(dioxane) were irradiated with a 532 nm laser beam using a confocal microscope system which enabled numerically controlled precision photocarving of the crystal surface. The changes to the cocrystal surface were subsequently characterized using scanning electron microscopy (SEM) (Figure 3, Supplementary Figure 12).

Upon irradiation with laser light of higher power (>5 mW, which corresponds to a light intensity of ∼5 W mm^–2^), larger holes of approximately 20 μm diameter were carved into the crystal surface, with SEM imaging revealing a significant buildup of material, most likely *trans*-**azo II**, with ridges over 1 μm in height forming around the area of the hole. However, the use of a lower power (1 mW) laser beam in the confocal microscope photocarving experiment was found to decrease the cut diameter to approximately 1 μm, with SEM imaging revealing the appearance of small plate-like crystals aligned parallel to the crystal surface. The appearance and orientation of the crystallites indicate a high mobility of *trans*-**azo** molecules, and crystallization of *trans*-**azo II**, constrained in the direction of the laser movement. The demonstrated control of the cuts/holes produced by the confocal laser system suggests that despite being based on a process that involves both molecular (*cis* → *trans*) and supramolecular (loss of volatile coformer) transformations, the photocarving of (*cis*-**azo**)(dioxane) can achieve a similar level of precision as reported for the analogous *trans*-**azo** cocrystal.

Photocarving was also studied visually, using a 78 μm beam diameter laser at powers of 5–15 mW (2.1–6.3 W mm^–2^) and a high-speed camera operating at 1000 frames per second (fps) (Supplementary Figures 13–15). While irradiation at the low power setting of 5 mW resulted only in minor etching of the cocrystal surface accompanied by a color change, the higher irradiation powers of 10 and 15 mW both resulted in photocarving, clearly evident by the formation of a hole in the crystal surface (Supporting Information Videos 3, 4, and 5). In each case irradiation led to the emission of a “fog” from the spot of the irradiation, which was assigned to the release of dioxane.^37^

### Photoresponsive Behavior of the (*cis*-**azo**)(pyrazine) Cocrystal

**Figure 4:**
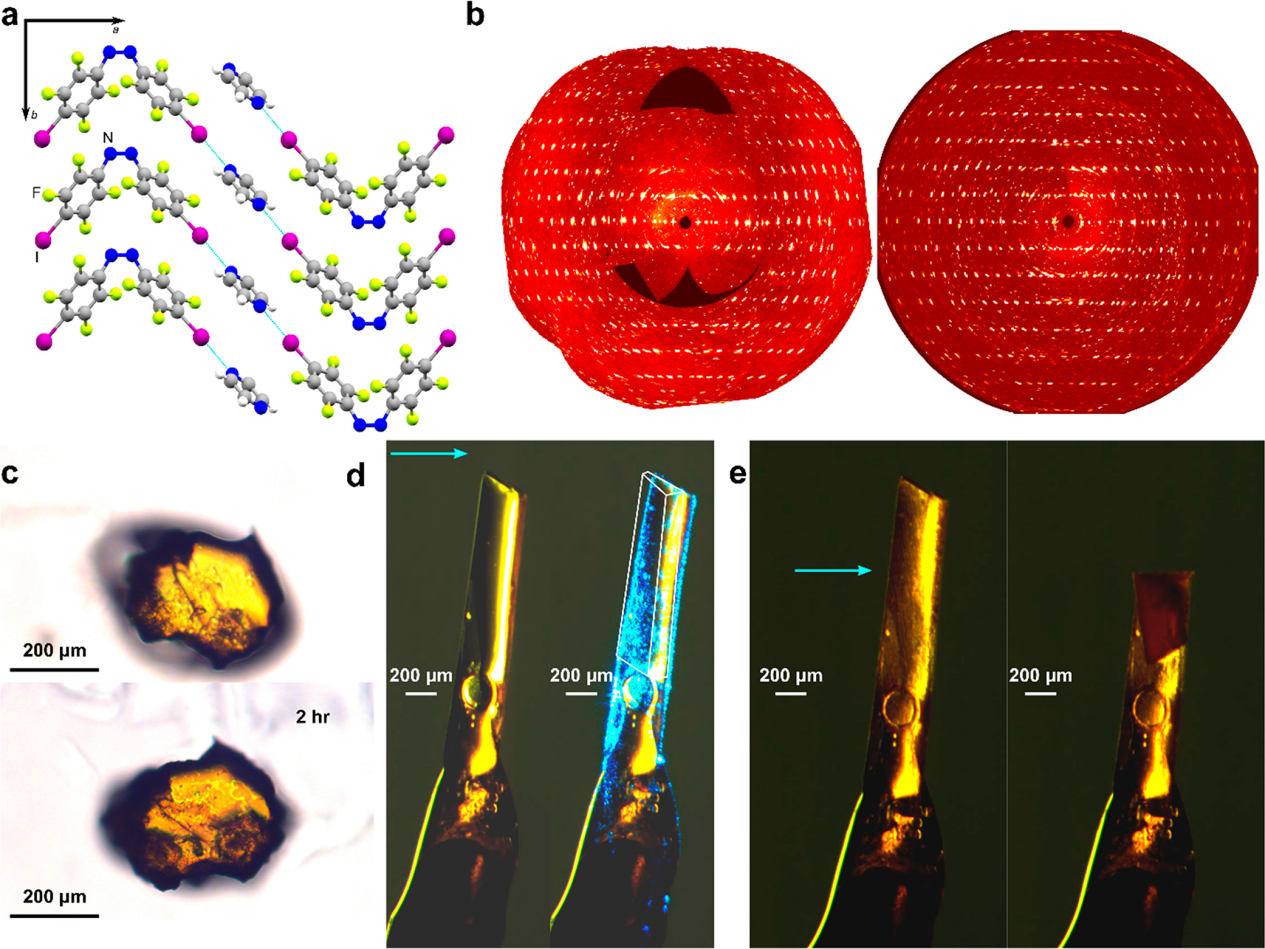
Overview of the photoresponsive behavior of the (*cis*-**azo**)(pyrazine) cocrystal. (a) Fragment of the crystal structure of the (*cis*-**azo**)(pyrazine) cocrystal, viewed along the crystallographic [001] direction. (b) Composite X-ray diffraction images for the crystallographic *h*0*l* plane for a (*cis*-**azo**)(pyrazine) single crystal before (left) and after (right) photocarving using a 532 nm, 2.25 W·mm^–2^ laser. After the first diffraction experiment the crystal was cut in half and the X-ray diffraction experiment was repeated on the remaining bottom half of the crystal. (c) Optical images of a (*cis*-**azo**)(pyrazine) crystal irradiated by dispersed 532 nm LED light. (d) Photographic images of a (*cis*-**azo**)(pyrazine) crystal that was irradiated with a 488 nm laser of 150 mW·mm^–2^, demonstrating photomechanical behavior with the first panel showing the crystal prior to irradiation and the second panel showing the crystal after 1 min of irradiation. The blue arrow represents the direction of the incident laser beam, and the white outline has been added to illustrate the positioning of the crystal prior to photomechanical bending. (e) Photographic images of a (*cis-***azo**)(pyrazine) crystal before (left) and after (right) being irradiated with a 532 nm, 2.25 W·mm^–2^ laser, resulting in cutting of the crystal.

Crystals of (*cis***-azo**)(pyrazine) were readily obtained by the crystallization of a preground equimolar mixture of *trans*-**azo** and pyrazine. The sample was then dissolved in hexanes with dropwise addition of CH_2_Cl_2_ until complete dissolution. The resulting solution was irradiated by a 532 nm LED source to induce the formation of *cis*-**azo** and left to crystallize by slow evaporation. Single crystal X-ray diffraction analysis (Figure 4a,b, also Supplementary Figure 1) revealed a structure analogous to that of (*cis***-azo**)(dioxane), with zigzag chains of alternating molecules of pyrazine and *cis*-**azo** connected through I···N halogen bonds. The I···N halogen-bonding distances were found to be slightly longer (*d*_I···N_RB = 2.896(5) Å, RXB = 0.820) than those in the previously reported (*trans*-**azo**)(pyrazine) cocrystal (*d*_I···N_ = 2.840(3) Å, RXB = 0.805).^39^ In contrast to needle-shaped crystals obtained with (*cis***-azo**)(dioxane), the crystals of (*cis***-azo**)(pyrazine) exhibit the form of large plates (typical dimensions 1000 × 250 × 50 μm^3^, Figure 4c–e).

The (*cis***-azo**)(pyrazine) cocrystal also exhibited three distinct types of responses upon irradiation with green or blue light of different light intensities. Irradiation with low-intensity 532 nm LED light led to a change in color from yellow to orange (Figure 4c), along with minor cracking of the crystal, attributed to the strain of *cis*–*trans* isomerization. After dissolving the irradiated cocrystal in CDCl_3_, ^19^F NMR analysis revealed that *cis*- to *trans*-**azo** conversion took place to an extent of ∼8% (Supplmentary Figure 16). Notably, such conversion was achieved after 2 h of irradiation, indicating greater photostability of (*cis*-**azo**)(pyrazine) compared to (*cis*-**azo**)(dioxane) which showed similar conversion after only 20 min of irradiation.

The (*cis*-**azo**)(pyrazine) crystals also exhibited a photomechanical response, albeit less pronounced than (*cis*-**azo**)(dioxane) (Figure 4d). Upon irradiation by dispersed laser light of 488 or 532 nm wavelength and intensity of 150 mW mm^–2^, a crystal of (*cis*-**azo**)(pyrazine) was observed to bend away from the source of irradiation over the course of a minute, with the irradiated section changing color from yellow to red, consistent with *cis* → *trans* isomerization (Supporting Information Video 6, shown at 4× speed).

Cold photocarving was demonstrated by exposure to either green or blue light, on both fresh or previously irradiated crystals. Using a sample of (*cis*-**azo**)(pyrazine), the sample was exposed to 532 nm laser radiation, showing the ability to readily carve through the crystal using a higher light intensity beam of 2.25 W mm^–2^ (Figure 4e, also Supporting Information Video 7). In contrast to their dioxane counterpart, crystals of (*cis*-**azo**)(pyrazine) were found to retain crystallinity after cutting with a 532 nm laser beam, which was evident from full X-ray diffraction data that were collected from a parent (*cis*-**azo**)(pyrazine) single crystal before irradiation and on the daughter crystal resulting from cutting (Figure 4b, Supplementary Figures 17 and 18). The freshly laser-cut crystal exhibited only X-ray diffraction signals of (*cis*-**azo**)(pyrazine), with the only indication of *cis* → *trans* isomerization being a slight change in the color of the cut crystal surface from yellow to red.

Changes to (*cis*-**azo**)(pyrazine) upon irradiation were also followed by PXRD (Supplementary Figure 19), by irradiating a bulk crystalline powder of the cocrystal with dispersed green (532 nm wavelength) LED light of light intensity 37 mW·cm^–2^. Visual inspection of the PXRD patterns of the material before and after irradiation indicated the complete transformation of (*cis*-**azo**)(pyrazine) to a physical mixture of solids *trans-***azo I** and **II** over a period of 20 h, demonstrating improved photostability compared to (*cis*-**azo**)(dioxane). Similar to (*cis*-**azo**)(dioxane), analysis of the PXRD patterns did not reveal any sign of (*trans*-**azo**)(pyrazine) or solid *cis*-**azo**, suggesting that *cis* → *trans* isomerization and loss of coformer upon irradiation of the (*cis*-**azo**)(pyrazine) cocrystal either are simultaneous or occur through intermediates that are too minute to be observed via PXRD. Attempts to follow the irradiation of (*cis*-**azo**)(pyrazine) by single crystal Raman spectroscopy were not successful due to cocrystals exhibiting significant fluorescence upon irradiation with the 785 nm laser probe.

Analysis by SEM revealed similar precision cuts compared to the analogous dioxane cocrystal (Supplementary Figure 20). Irradiation of a (*cis*-**azo**)(pyrazine) crystal with a higher power green laser beam (>15 mW) after 5 s produced a hole at the spot of irradiation, with significant material buildup around the hole. Reducing the power of the laser line (to ∼3 mW) and automating a linear cutting process revealed plate-like crystallites forming on the edge of the cut similar to those seen for (*cis*-**azo**)(dioxane), again indicating displacement from the irradiated area and crystallization of *trans*-**azo II**.

### Theoretical Studies

The stability of *cis*-**azo** cocrystals was also evaluated and compared to that of the corresponding *trans*-**azo** systems through calculations based on periodic density-functional theory (DFT) with semiempirical dispersion correction (SEDC). Specifically, periodic DFT was used to calculate the energy difference (Δ*E*_iso_, Table 1) between (*cis*-**azo**)(dioxane) or (*cis*-**azo**)(pyrazine) and their *trans*-**azo** analogs,^37^ as well as the energy of cocrystal decomposition (Δ*E*_dec_, Table 2) into the crystalline solid **azo** and the gaseous coformer. The Δ*E*_dec_ values were calculated for two possible outcomes of cocrystal decomposition, specifically the formation of the **azo** component as either the solid *cis*-**azo** or the *trans*-**azo** polymorph **II**. Finally, the strength of halogen bonding (*E*_XB_)) in each cocrystal was evaluated by calculating the interaction strength for a pair of **azo** and coformer molecules (Table 2).

**Table 1:** Calculated eEnergy of *cis* → *trans* Isomerization for the Cocrystals and **azo** Components

cocrystal isomerization process	Δ*E*_iso_, kJ mol^–1^
(*cis*-**azo**)(dioxane) → (*trans-***azo**)(dioxane)	–34.25
(*cis*-**azo**)(pyrazine) → (*trans-* **azo**)(pyrazine)	–43.68
*cis*-**azo** → *trans-***azo I**	–37.67
*cis*-**azo** → *trans-***azo II**	–36.97
*cis*-**azo** → *trans-***azo** (g)^19^	–36.99

**Table 2:** Calculated Energies (in kJ mol^–1^) Associated with the Loss of Volatile Coformer from the Crystal Structures of (*cis*-**azo**)(dioxane), (*cis*-**azo**)(pyrazine), (*trans*-**azo**)(dioxane), and (*trans*-**azo**)(pyrazine) Cocrystals, and Energy of Halogen Bond (XB) Interactions

cocrystal	Δ*E*_dec_(*cis*), kJ mol^–1^	Δ*E*_dec_(*trans* II), kJ mol^–1^	*E*_XB_, kJ mol^–1^
(*cis*-**azo**) (dioxane)	86.86	49.74	–18.55
(*cis*-**azo**) (pyrazine)	100.18	63.07	–28.11
(*trans*-**azo**) (dioxane)	N/A	84.13	–19.05
(*trans*-**azo**) (pyrazine)	N/A	106.86	–27.15

The calculations indicated that the isomerization of the *cis*- to the respective *trans*-**azo** cocrystal should be energetically favorable in all cases, with Δ*E*_iso_ values of −34.25 and −43.68 kJ·mol^–1^ for the (*cis*-**azo**)(dioxane) and (*cis*-**azo**)(pyrazine) cocrystal, respectively. These energy differences are also in good agreement with those calculated for individual crystalline **azo**-compounds, e.g., isomerization of *cis*-**azo** to either *trans*-**azo I** (−37.67 kJ mol^–1^) or *trans*-**azo II** (−36.97 kJ mol^–1^), and with previously reported gas-phase calculations^19^ (−36.99 kJ mol^–1^). The significant stabilization calculated for the process of *cis* → *trans* isomerization is in agreement with Raman spectroscopy, which indicated that the isomerization takes place prior to coformer evaporation. The calculated values indicate that the *cis* → *trans* isomerization in the (*cis*-**azo**)(pyrazine) cocrystal is ∼10 kJmol^–1^ more favorable compared to (*cis*-**azo**)(dioxane).

Decomposition energies for the cocrystals were calculated considering two potential decomposition pathways: the *cis*-**azo** cocrystals converting either into a mixture of crystalline *cis*-**azo** and the gaseous coformer or into a mixture of crystalline *trans*-**azo II** and gaseous coformer. The calculated values suggest that both decomposition pathways should be energetically unfavorable, which is consistent with the required input of energy in the form of light or heat. Independent of the decomposition pathway, calculations indicate that the pyrazine-based cocrystals should be more stable against decomposition than their dioxane counterparts, which is in agreement with the results of the irradiation experiments. The calculated halogen-bonding energies additionally highlight the stability of the pyrazine cocrystals compared to their dioxane counterparts and suggest that the increase in halogen-bonding strength upon switching from an oxygen-based to a nitrogen-based acceptor contributes to the overall stability of the cocrystal.

### Thermal Analysis

**Figure 5:**
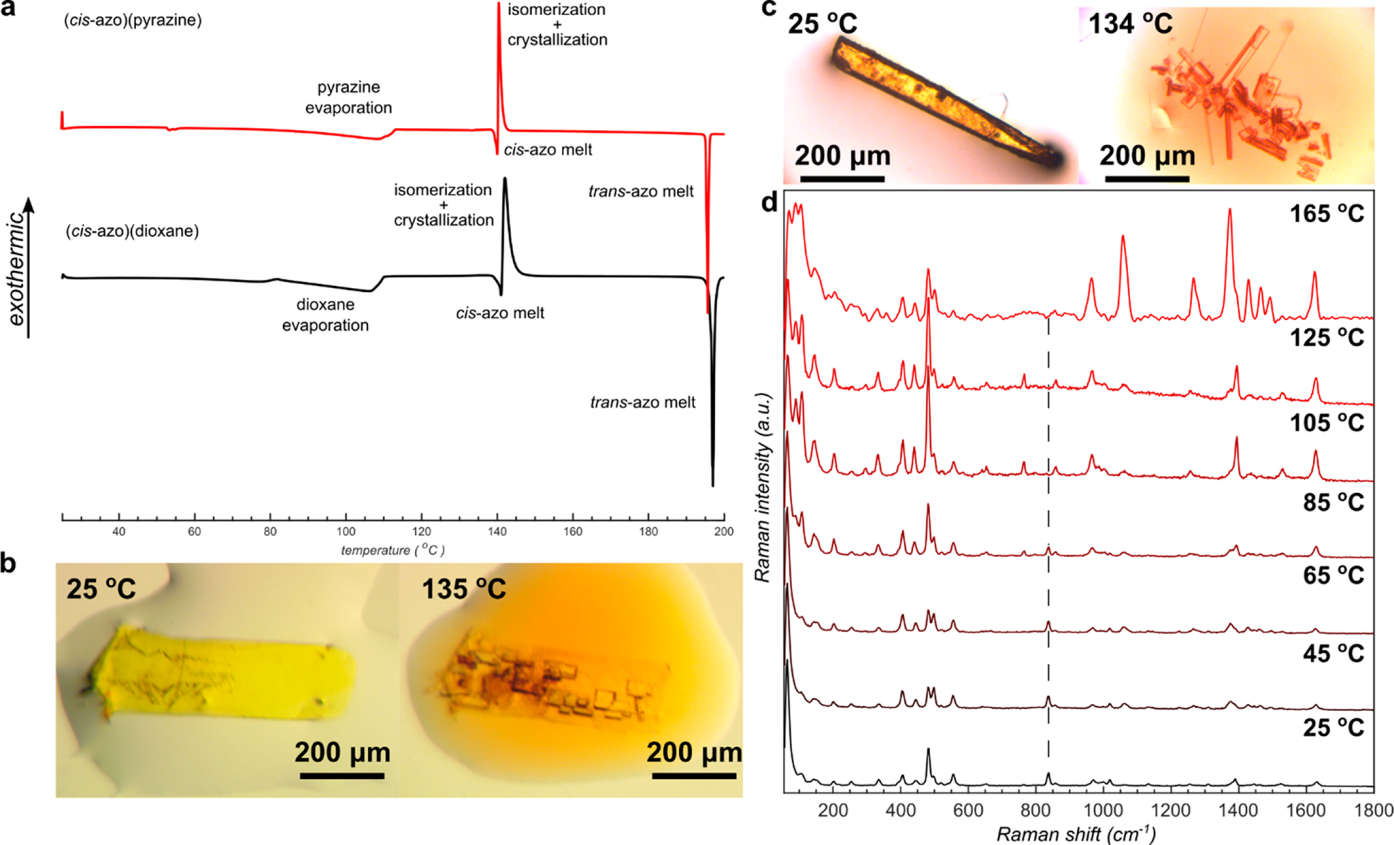
Thermal studies of the *cis*-azo cocrystals. (a) Overlay of DSC thermograms for the (*cis-***azo**)(pyrazine) (red) and (*cis*-**azo**)(dioxane) (black) cocrystals. The weak endothermic signal around 52 °C in the (*cis-***azo**)(pyrazine) thermogram corresponds to the melting point of a small amount of solid pyrazine present. (b) Hot-stage optical microscopy images of a (*cis*-**azo**)(pyrazine) crystal in paraffin oil, before and after recrystallization into *trans-***azo**. (c) Hot-stage optical microscopy images of a (*cis*-**azo**)(dioxane) in paraffin oil, before and after recrystallization into *trans-***azo**. (d) Overlay of Raman spectra collected from a single crystal of (*cis*-**azo**)(dioxane) at different temperatures demonstrating the desolvation of the dioxane prior to isomerization into *trans*-**azo**, the dashed line representing the location of the Raman band for the ring-breathing vibration of dioxane.

The observed mechanism of light-driven isomerization and loss of coformer from (*cis*-**azo**)(dioxane) is different from the analogous heat-driven process, as evidenced by differential scanning calorimetry (DSC) and thermogravimetric analysis (TGA) on a bulk sample of (*cis*-**azo**)(dioxane). The DSC thermogram for (*cis*-**azo**)(dioxane) heated at a rate of 5 C° min^–1^ shows an endothermic process in the 80–110 °C range, attributed to dioxane loss (Figure 5a). This assignment was supported by TGA, which revealed a 14.2% loss of weight in the same thermal range, consistent with the theoretically calculated content of dioxane (13.2%) in (*cis*-**azo**)(dioxane) (Supplementary Figure 21). Further heating leads to another endothermic event at 141 °C, immediately followed by an exothermic process, which has been interpreted as the melting of *cis*-**azo** followed by thermally induced isomerization and crystallization into solid *trans*-**azo**.^32^ Upon further heating, the DSC thermogram exhibits a sharp endothermic process at 197 °C, consistent with the melting of *trans*-**azo**. The interpretation of DSC data was verified by hot-stage optical microscopy (Figure 5c, Supplementary Figure 25). Heating of a yellow (*cis*-**azo**)(dioxane) crystal did not produce visible changes in shape or color until 104 °C, when the crystal begins to darken and crack, which was interpreted as due to the loss of dioxane coformer. Indeed, if the heating is conducted with a (*cis*-**azo**)(dioxane) cocrystal submerged in oil, the darkening of the crystal is associated with the appearance of bubbles on the crystal surface, consistent with the removal of gaseous dioxane. Further heating leads to liquefaction at ∼121 °C, followed by recrystallization at ∼139 °C to form crystallites of *trans*-**azo** that subsequently melt at the expected melting point temperature of 185 °C. Thermal decomposition of (*cis*-**azo**)(dioxane) was also studied *in situ* through variable-temperature Raman spectroscopy (Figure 5d) on a single crystal of (*cis*-**azo**)(dioxane). While the initial Raman spectrum (25 °C) revealed only bands of *cis*-**azo** and dioxane (ν(C–O–C) at 830 cm^–1^), increasing the temperature to 105 °C led to the appearance of new low-frequency bands (<150 cm^–1^), indicating changes in intermolecular vibrations, and the disappearance of the dioxane Raman band at 830 cm^–1^. Further heating to 165 °C leads also to the appearance of new Raman bands that were consistent with *trans*-**azo**. These observations are consistent with the thermal decomposition of (*cis*-**azo**)(dioxane) proceeding first through the loss of dioxane, followed by *cis* → *trans* isomerization.

Overall, the sequence of heat-induced events that transform (*cis*-**azo**)(dioxane) into *trans*-**azo** is clearly different from the analogous light-driven process. The DSC, TGA, and hot-stage microscopy show that the first step in the heat-driven process is desolvation of the cocrystal to form solid *cis*-**azo** which subsequently melts and then undergoes isomerization and crystallization to form solid *trans*-**azo**. In contrast, irradiation of (*cis*-**azo**)(dioxane) proceeds through a qualitatively distinct process of *cis* → *trans* isomerization to first yield solid (*trans*-**azo**)(dioxane) which subsequently loses dioxane to form solid *trans*-**azo**.

The DSC analysis of bulk (*cis*-**azo**)(pyrazine) (Figure 5a) revealed three distinct endothermic events, the first between 80 and 115 °C, which was identified as loss of pyrazine coformer (confirmed by TGA, which revealed a mass loss of ∼12.5%, Supplementary Figure 24), the second at 140 °C, which was assigned as *cis*-**azo** melt, and the third was at 196 °C, which was assigned to the melting of *trans*-**azo**. An exothermic process was also observed between approximately 140 and 145 °C, which was identified as a combination of *cis* → *trans* isomerization and recrystallization. The interpretation of DSC measurements was again validated by variable temperature optical microscopy on a single crystal of (*cis*-**azo**)(pyrazine), which was conducted both with a neat crystal (Supplementary Figure 26) and with a crystal submerged in oil (Figure 5b). The two experiments yielded very similar results, with the crystal retaining the overall appearance until ∼90 °C, followed by darkening and the appearance of bubbles (in the case of the crystal submerged in oil) at higher temperatures. These observations are consistent with *cis* → *trans* isomerization and thermal loss of pyrazine. After ∼140 °C the sample consists of solid *trans*-**azo**, which was identified by PXRD, and subsequently either melts or dissolves in oil at around 200 °C.

## Conclusion

We presented new cocrystal-based materials that exhibit multiple and different optical, mechanical, and structural responses to visible green or blue light. Using a high-intensity light beam, the herein presented halogen-bonded cocrystals can be readily carved, machined, or engraved. Upon reducing light intensity, however, the photoresponse can be switched from photocarving to photomechanical bending. Further reduction in the light intensity enables the photoresponse to be further modified, leading only to a change in crystal color. These various responses in a single material are enabled by the general cocrystal design which pairs a *cis*-azobenzene chromophore halogen-bonded to a volatile cocrystal former (dioxane, pyrazine). The light-induced color change and photomechanical bending occur due to different extents of the dye *cis* → *trans* isomerization taking place at different irradiation intensities, while the photocarving effect is due the combination of the volatility of the cocrystal former and the photochromic nature of the azobenzene. Mechanistic studies by *in situ* Raman spectroscopy on a single crystal show that light-driven desolvation of the (*cis*-**azo**)(dioxane) cocrystal occurs through an intermediate of (*trans*-**azo**)(dioxane), i.e., that light absorption leads first to isomerization, followed by loss of volatile cocrystal component. By conducting analogous thermal analysis and hot-stage microscopy experiments, this mechanism is shown to be different from the analogous heat-induced process, in which cocrystal decomposition is observed prior to the isomerization of the **azo** component. Overall, this work demonstrates the ability to design multiresponse organic crystals by combining light-responsive behavior at the molecular (*cis* → *trans* isomerization) and supramolecular (light-induced loss of volatile coformer) levels. We believe that this simple design for dye-volatile cocrystals provides an exciting new opportunity to rationally design materials that not only respond to an external stimulus but also can adapt their response to finer properties of the stimulus.

## Experimental/Methods

### Synthesis

The compound *trans*-**azo** was synthesized in one step by treatment of 4-iodo-2,3,5,6-tetrafluoroaniline with *N*-chlorosuccinimide (NCS) and 1,8-diazabicyclo[5.4.0]undec-7-ene (DBU).^52^ All reagents and solvents were purchased from Sigma-Aldrich. For cocrystallization of (*trans*-**azo**)(dioxane) approximately 6 mg of *trans*-**azo** (0.010 mmol) was dissolved in a minimal volume of 1,4-dioxane (∼2 mL). The solution was then irradiated for 30 min via a 532 nm 37 mW·cm^–2^ LED and left to evaporate at room temperature, yielding long, yellow, needle-shaped crystals. For cocrystallization of (*cis*-**azo**)(pyrazine), approximately 6 mg of *trans*-**azo** (0.010 mmol) and 6 mg of pyrazine (0.075 mmol) were mixed in hexanes (∼5 mL) with methylene chloride added dropwise until all material was fully dissolved. The solution was then irradiated with a 532 nm 37 mW·cm^–2^ LED. The solution was left to evaporate at room temperature, yielding lath-shaped yellow crystals.

### Powder X-ray Diffraction

Powder X-ray diffraction experiments were performed on a Bruker D8 Advance diffractometer, using Cu Kα radiation (λ = 1.541 84 Å) source operating at 40 mA and 40 kV, equipped with a Lynxeye XE linear position-sensitive detector, in the 2θ range of 4–40° with a step size of 0.019° or, alternatively, on a Bruker D2 Phaser diffractometer using nickel-filtered Cu Kα radiation (λ = 1.541 84 Å) source operating at 10 mA and 30 kV, equipped with a Lynxeye linear position-sensitive detector, in the 2θ range of 4–40°.

### Single-Crystal X-ray Diffraction

Data for (*cis*-**azo**)(dioxane) and (*cis*-**azo**)(pyrazine) were collected on a Bruker D8 Venture dual-source diffractometer equipped with a PHOTON II detector and an Oxford Cryostream 800 cooling system, using mirror-monochromated Mo Kα (λ = 0.710 73 Å) or Cu Kα radiation (λ = 1.541 84 Å) from respective microfocus sources. Data were collected in a series of φ- and ω-scans. APEX3 software was used for data collection, integration, and reduction.^53^ Numerical absorption corrections were applied using SADABS-2016/2.^54^ Structures were solved by dual-space iterative methods using SHELXT^55^ and refined by full-matrix least-squares on *F*^2^ using all data with SHELXL,^56^ within the OLEX2,^57^ and/or WinGX^58^ environments.

### UV–Visible Absorbance Spectroscopy

Absorbance measurements were collected on an Agilent Cary 300 Bio UV–visible spectrometer. A 55 mg/L solution of *trans*-**azo** was prepared in THF, and the absorbance spectrum was acquired using instrument default conditions. The spectrum of the corresponding *cis*-isomer was collected following 30 min of irradiation by a 532 nm LED (37 mW).

### Raman Spectroscopy

Raman microscopy experiments were performed on a confocal Raman Witec 300 R microscope setup using two separate probe wavelengths of 785 and 532 nm. Integration time, number of accumulations, and laser power were varied depending on the experiment. Simulated Raman shifts were calculated by DFT using Gaussian 16,^59^ employing the B3LYP density functional.^60,61^ Basis sets 6-311G(d,p) were used for all atoms.^62^ The 6-311G(d,p) basis set parameters for iodine^63^ were obtained from the Basis Set Exchange.^64^

### Thermal Analysis

Thermogravimetric analysis (TGA) and differential scanning calorimetry (DSC) measurements were performed simultaneously using a Mettler-Toledo TGA/DSC 1 Star system thermobalance. The samples were placed in alumina crucibles, and measurements were conducted under a stream of nitrogen (50 cm^3^ min^–1^) gas, at a heating rate of 5 °C min^–1^. Data collection and analysis were performed using the Mettler-Toledo STAR^e^ software 16.20 program package. Alternatively, DSC measurements were performed on a TA Instruments LTD DSC2500 at a heating rate of 1 °C min^–1^, under a stream of nitrogen (50 cm^3^ min^–1^) gas, using a hermetically closed aluminum pan. Hot-stage microscopy was performed using a Mettler Toledo FP90 central processor, equipped with a Mettler FP84 HT TA microscopy cell. Images were obtained on an Infinity 1 Lumenera camera attached to a Leica DM2500 optical microscope, using the Studio Capture software suite. Heating was performed from 20 to 200 °C at a rate of 20 °C min^–1^.

### Scanning Electron Microscopy

Single crystals of (*cis*-**azo**)(dioxane) and (*cis*-**azo**)(pyrazine) were sputter-coated with palladium and placed into an FEI Helios Nanolab 660 DualBeam (focused ion beam-extreme high-resolution scanning electron microscope) for imaging.

### Detailed Machining Procedure

A confocal Raman Witec 300 R 532 nm solid-state laser was used with a range of power settings (0.1–20 mW) and multiple objectives (10, 20, 50, and 100× Zeiss objectives with NA = 0.25, 0.5, 0.8, and 0.9, respectively). A series of detailed drawings were copied and then engraved into the crystals using a premade input file with listed coordinates.

### Laboratory Laser System Including a High-Speed Camera

A Redlake MotionPro Y4 (Tallahassee, FL, USA) high-speed charge-coupled device camera was used to capture machining events at 1000 frames/s. A MGL-III-532 Green DPSS laser and Lasos Lasertechnik 633 nm helium–neon laser was coupled with a tunable neutral density filter, a Melles Griot electronic controlled shutter, and a Melles Griot convex lens with a focal length of 75 mm, aligned onto a crystal mount with a 150 μm loop (Supplementary Figure 4).

### Periodic DFT Calculations

Periodic DFT calculations were performed using the plane-wave DFT code CASTEP.^65^ The input files were prepared from the CIFs of the experimentally determined crystal structures using the program cif2cell.^66^ Crystal structures of all materials were geometry-optimized with respect to atom positions and unit cell parameters subject to space group symmetry constraints. The calculations were performed with PBE^67^ functional combined with a Grimme D3 semiempirical dispersion correction scheme.^68^ The plane-wave basis set was truncated at 750 eV cutoff, and the first electronic Brillouin zone was sampled with the 2π × 0.07 Å^–1^ Monkhorst–Pack grid k-point spacing.^69^ Tight convergence criteria were used in the optimization, namely, 5 × 10^–6^ eV atom^–1^ for total energy, 0.01 eV Å^–1^ for atomic forces, 5 × 10^–4^ Å for atomic displacement, and 0.02 GPa for residual stress.

Calculations for the gas-phase molecules were performed by placing one molecule in a cubic simulation cell of 20 Å dimensions and performing a fixed cell geometry optimization. In that case, a single Γ k-point was used for electronic Brillouin zone sampling; all other settings were kept the same as for the optimization of the crystal structures. For dimer interaction energies, two molecules were placed in the same 30 Å × 30 Å × 30 Å simulation box with their geometries extracted from the optimized crystal structures. In that case, only single-point calculations were performed rather than geometry optimizations, since the aim was to calculate interaction energies for the in-crystal intermolecular orientation. The dimer interaction energies were computed by subtracting the total energies of individual molecules from the total energy of the dimer. The energies of individual crystal structures and gas-phase molecules were used to compute the decomposition energies.

### NMR Spectroscopy

Spectra shown in Supplementary Figures 7, 8, and 16 were acquired on a Bruker Avance NEO 400 MHz spectrometer equipped with a BBFO probe. The ^19^F *T*_1_ was measured on a sample containing both *cis*- and *trans* isomers of the (**azo**)(dioxane) cocrystal dissolved in CDCl_3_ and found to be 2.0 ± 0.2 s for both isomers, for the signals of ^19^F atoms closest to −118 ppm (in ortho-position to the iodine atom). The spectra were acquired with a recycling delay of 10 s. A 90°–180°–90° background suppression sequence was used to remove probe background and irradiation was centered at −130 ppm.^70^

The spectra shown in Supplementary Figure 10 were acquired on a Bruker 400 MHz spectrometer (376 MHz for ^19^F NMR, with an AVANCE Neo console using a BBFO probe) and are reported in ppm. For ^19^F NMR, a relaxation delay (D1) of 10 s was used, with a pulse angle of 90° to maximize SNR. 8000 scans and 10 000 scans were collected over a spectral width of −300 to 100 ppm, and the Bruker inverse-gated ^1^H decoupled pulse sequence (zgig) was used.

## Supporting Information Available

Details of experiment and instrument designs, theoretical methodology, selected NMR, infrared and Raman spectroscopy, powder and single crystal X-ray diffraction, electron microscopy data, thermal microscopy, and high-speed optical recording data (PDF)
Video 1 showing photomechanical bending of (*cis*-**azo**)(dioxane) produced at 4× speed (MP4)
Video 2 showing photocarving of (*cis*-**azo**)(dioxane) (MP4)
Video 3, high-speed camera video of (*cis*-**azo**)(dioxane) photocarving, 15 mW, 532 nm irradiation (MP4)
Video 4, high-speed camera video of (*cis*-**azo**)(dioxane) photocarving, 10 mW, 532 nm irradiation (MP4)
Video 5, high-speed camera video of (*cis*-**azo**)(dioxane) photocarving, 5 mW, 532 nm irradiation (MP4)
Video 6 showing photomechanical bending of (*cis*-**azo**)(pyrazine) produced at 4× speed (MP4)
Video 7 showing photocarving of (*cis*-**azo**)(pyrazine) (MP4)
