# Supplementary material for: Three-in-One: Dye-Volatile Cocrystals Exhibiting Intensity-Dependent Photochromic, Photomechanical, and Photocarving Response
Source: J Am Chem Soc. 2023 Nov 4;145(45):24636–47. doi: 10.1021/jacs.3c07060 (PMC10655124; doi:10.1021/jacs.3c07060)
Supplement: Supplementary file 1 — ja3c07060_si_001.pdf [file ja3c07060_si_001.pdf]

Supplementary Information for:

Three-in-one: dye-volatile cocrystals exhibiting intensity-dependent photo-chromic, photo-mechanical, and photo-carving response

Tristan H. Borchers,[1,2] Filip Topić,[1] Mihails Arhangelskis,[3] Jogirdas Vainauskas,[1,2] Hatem M. Titi,[1]

Oleksandr S. Bushuyev,[1] Christopher J. Barrett,[1] Tomislav Friščić[1,2]**

[1]Department of Chemistry, McGill University, Montreal, Canada. *christopher.barrett@mcgill.ca

[2]School of Chemistry, University of Birmingham, Birmingham, United Kingdom. *t.friscic@bham.ac.uk

[3]Faculty of Chemistry, University of Warsaw, Warsaw, Poland.

Table of Contents

1. Single crystal X-ray diffraction p2 – p4
2. UV-Vis absorbance p5
3. Laboratory laser setup p5 – p7
4. (*cis*-**azo**)(dioxane) cocrystal p8 – p14
   4.1. NMR analysis of (*cis*-**azo**)(dioxane) p8 – p10
   4.2. Raman spectroscopy p11 – p12
   4.3. Scanning electron microscopy p12
   4.4. High-speed camera studies p13 – p14
5. (*cis*-**azo**)(pyrazine) cocrystal p14 – p18
   5.1. NMR analysis of (*cis*-**azo**)(pyrazine) p14
   5.2. Crystallographic studies of photo-carving of single crystals p15
   5.3. X-ray diffraction analysis p16
   5.4. Scanning electron microscopy p17
   5.5. Computational analysis of cocrystals p17 – p18
6. Thermal analysis p18 – p20
7. Additional photo-carving images and videos p21 – p22
   7.1. Supplementary video and data file information p22
8. References p23

1. Single crystal X-ray diffraction:

The X-ray data for (*cis*-**azo**)(dioxane), (*cis*-**azo**)(pyrazine) were collected on a Bruker D8 Venture dual-source diffractometer equipped with a PHOTON II detector and an Oxford Cryostream 800 cooling system, using mirror-monochromated Mo*K*α ($\lambda = 0.71073$ Å) or Cu*K*α radiation ($\lambda = 1.54184$ Å) from respective microfocus sources. Data were collected in a series of φ- and ω-scans. APEX3 software was used for data collection, integration and reduction.[1] Numerical absorption corrections were applied using SADABS-2016/2.[2]

Structures were solved by dual-space iterative methods using SHELXT[3] and refined by full-matrix least-squares on F^2 using all data with SHELXL[4] within the OLEX2[5] and/or WinGX[6] environment. For both structure models, some reflections were found to have been obscured by the beam stop and were omitted from the refinement. Hydrogen atoms were placed in calculated positions and treated as riding on the parent carbon atoms with isotropic displacement parameters 1.2 times larger than the respective parent atoms. Equivalent 1,2- and 1,3-distances were restrained to be of the same length for dioxane molecules in (*cis*-**azo**)(dioxane), with rigid bond and proximity restraints also applied to their anisotropic displacement parameters. The dioxane molecules were modelled as disordered around the inversion centre over two components, with their occupancies refining to 0.675(10) and 0.325(10), respective.

Crystal structure figures were generated using Mercury,[7] and POV-Ray methods.[8]

CCDC 2162225 and 2162226 contain the supplementary crystallographic data for this paper. The data can be obtained free of charge from The Cambridge Crystallographic Data Centre *via* www.ccdc.cam.ac.uk/structures.

Supplementary Table 1. Crystallographic data for the reported crystal structures of (*cis*-**azo**)(dioxane) and (*cis*-**azo**)(pyrazine).

Compound	(*cis*-**azo**)(dioxane)	(*cis*-**azo**)(pyrazine)
CCDC Number	2162225	2162226
T (K)	253.0(1)	298(2)
Formula	$C_{16}H_8F_8I_2N_2O_2$	$C_{16}H_4F_8I_2N_4$
M_r	666.04	658.03
Crystal system	Monoclinic	Monoclinic
Space group	$C2/c$	$C2/c$
a (Å)	21.1357(14)	29.0978(6)
b (Å)	5.4973(4)	5.72450(10)
c (Å)	18.4971(12)	11.5731(3)
α (°)	90	90
β (°)	112.909(2)	95.6637(11)
γ (°)	90	90
V (Å^3)	1979.6(2)	1918.32(7)
Z	4	4
ρ_{calc} (g cm^{-3})	2.235	2.278
λ (Å)	0.71073	1.54184
μ (mm^{-1})	3.266	26.589
F(000)	1248.0	1224
Crystal size (mm^3)	0.522 × 0.120 × 0.075	0.456 × 0.214 × 0.096
Data collection θ range (°)	3.852–32.629	3.052–72.433
Reflections collected [R_{int}]	28174	14995
Reflections [$I > 2\sigma(I)$]	2915	1556
Data completeness (%)	98.9	99.4
Data/parameters/restraints	3596/164/103	1875/136/0
Goodness-of-fit on F^2	1.065	1.092
Final R, data with $I > 2\sigma(I)$	R_1 = 0.0313<br>wR_2 = 0.0737	R_1 = 0.0346<br>wR_2 = 0.0771
Final R for all data	R_1 = 0.0420<br>wR_2 = 0.0817	R_1 = 0.0460<br>wR_2 = 0.0882
Largest diff. peak/hole (e Å^{-3})	1.196/−1.015	0.856/−0.864

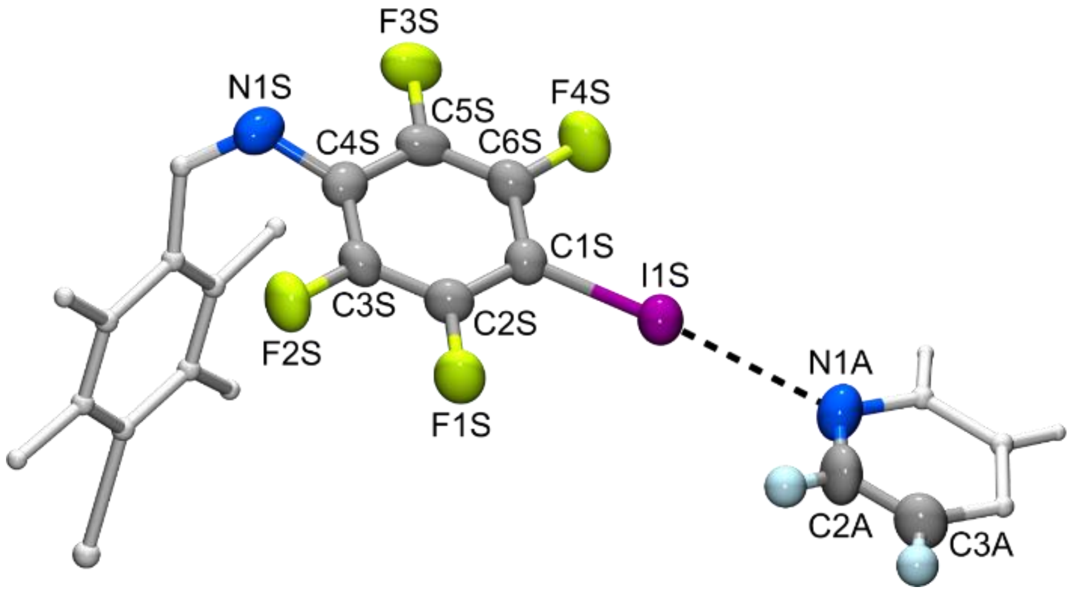

Supplementary Fig. 1. ORTEP view of the asymmetric unit of (*cis*-**azo**)(pyrazine), collected at 298 K. showing the atom labelling scheme (CCDC code). Hydrogen atoms are shown as small spheres of arbitrary radius. The symmetry-dependent parts of *cis*-**azo** and pyrazine molecules are shown in ball-and-stick model in light grey. The halogen bond is shown as a dashed black line.

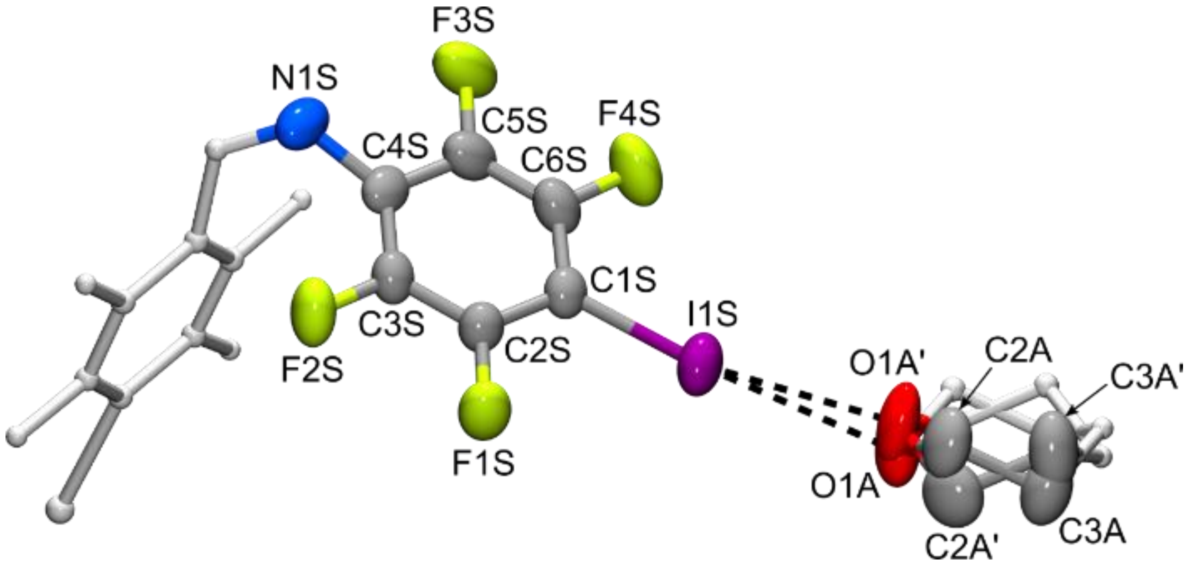

Supplementary Fig. 2. ORTEP view of the asymmetric unit of (*cis*-**azo**)(dioxane), collected at 253 K. showing the atom labelling scheme (CDC code). Displacement ellipsoids are drawn at a 50 % probability level. The symmetry-dependent parts of *cis*-**azo** and dioxane molecules are shown in ball-and-stick model in light grey. Halogen bonds are shown as a dashed black line.

2. UV-Vis absorbance:

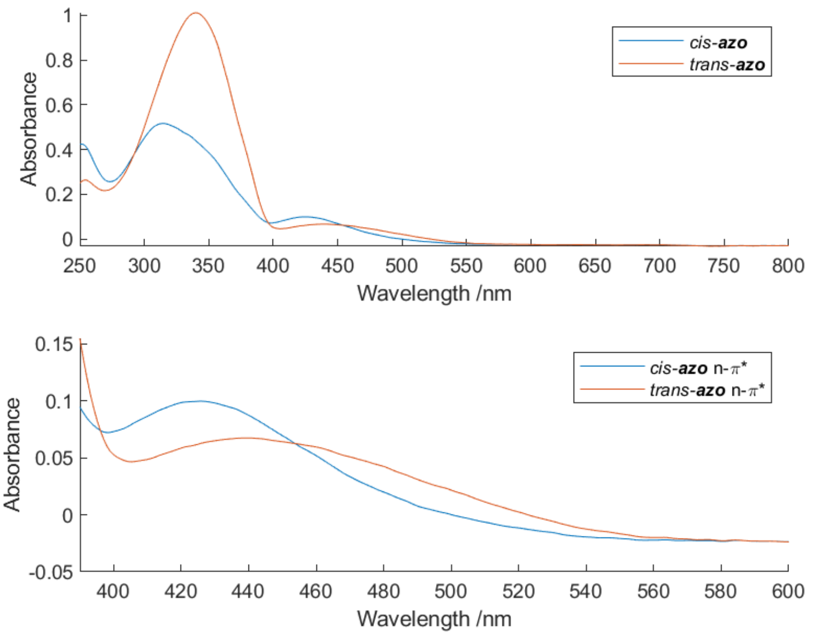

Supplementary Fig. 3. UV-Vis absorbance spectra of *trans*- and *cis*-**azo** in THF solutions.

3. Laboratory laser setup:

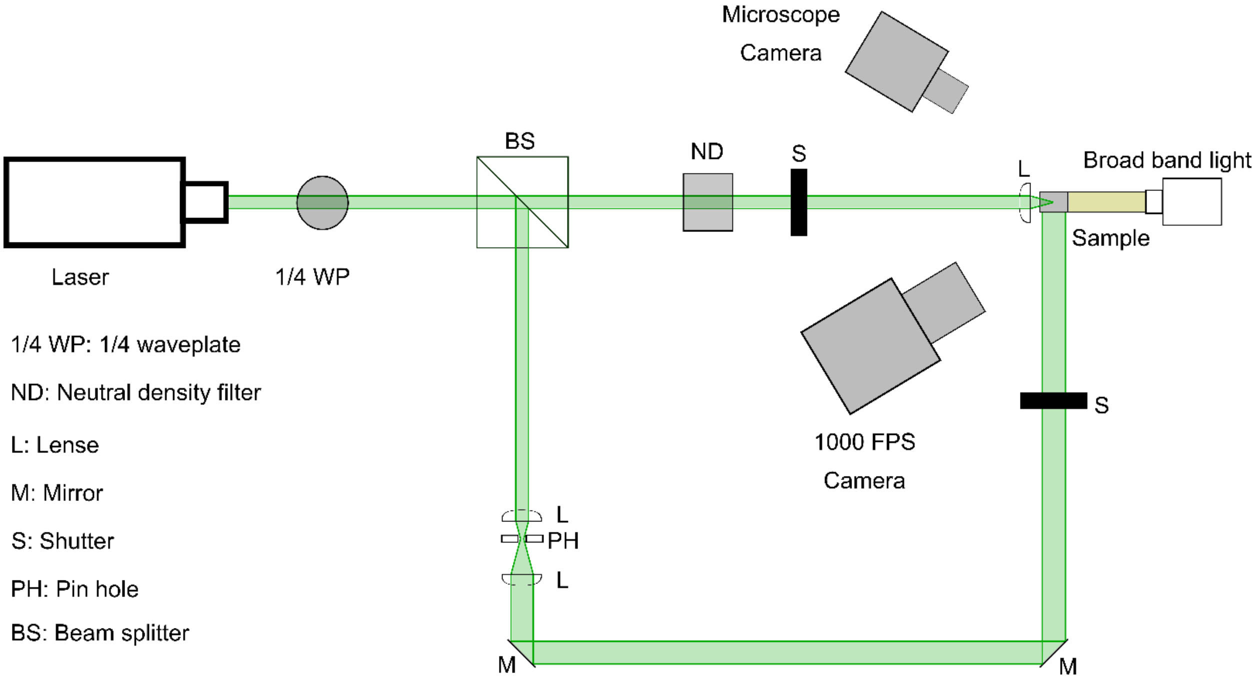

Supplementary Fig. 4. Schematic of the laboratory laser setup including the various optical components and equipment and multiple cameras.

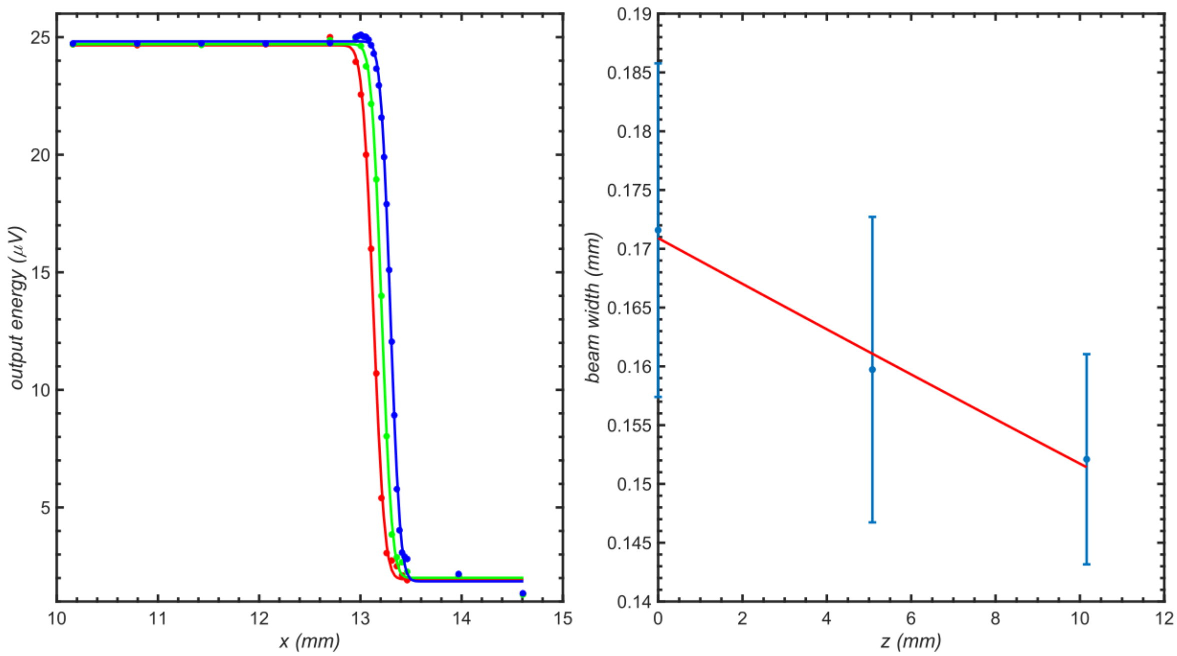

Supplementary Fig. 5. Determination of beam diameter at the irradiation surface of the photo-carving beam *via* a knife edge experiment. Left) output energy with respect to knife position. Right) beam width at various positions of sampling.

$$P = P_0 + \frac{P_{max}}{2}(1 - \mathrm{erf}\left(\frac{\sqrt{2}(x - x_0)}{w}\right))$$

Supplementary Formula 1. Fit for measured data, P_0 is background power, P_{max} is maximum power, x_0 is the position of shift at half of the real power, erf is a standard error function, and w is the beam radius.

$$f(x) = a \cdot e^{-(x-b)^2 / 2c^2}$$

Supplementary Formula 2. Gaussian function used to simulate the beam intensity profile where the variable a is the intensity at the centre of the beam diameter.

$$I_0 = \frac{2 \cdot P_0}{\pi \cdot {w_0}^2}$$

Supplementary Formula 3. intensity of a beam profile at the centre of the beam diameter; P_0 = total power of the beam, and w_0 = beam radius.

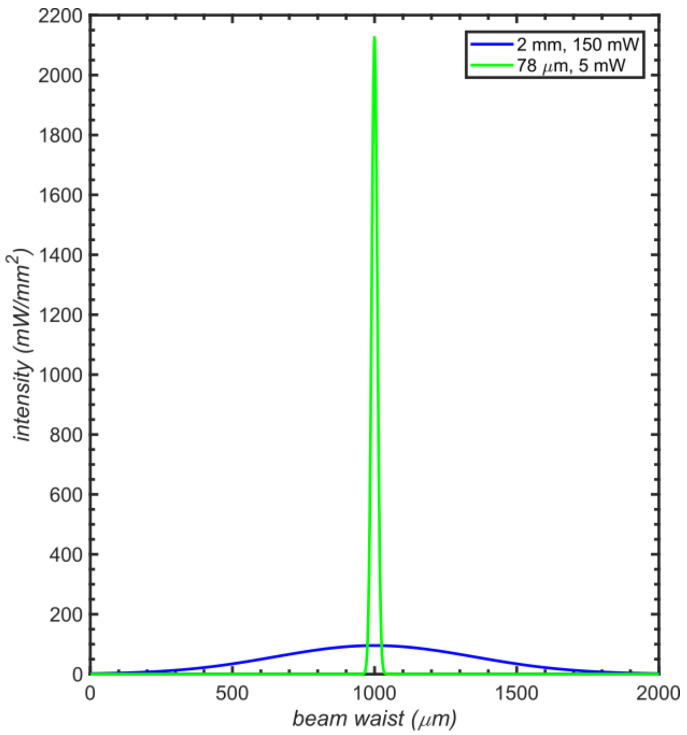

Supplementary Fig. 6. A Gaussian beam profile, to show the intensity distribution of the two beams used throughout this study; a photo-carving beam (green), and a photo-actuating beam (blue). The beam size of the photo-carving beam was calculated *via* a knife-edge experiment.

4. (*cis*-azo)(dioxane) cocrystal:

Spectra shown in Supplementary Figure S7, S8 and S16 were acquired on a Bruker Avance NEO 400 MHz spectrometer equipped with a BBFO probe. The ^{19}F T_1 was measured on a sample containing both *cis*- and *trans* isomers of the (**azo**)(dioxane) cocrystal dissolved in $CDCl_3$ and found to be 2.0±0.2 s for both isomers, for the signals of ^{19}F atoms closest to -118 ppm (in ortho-position to the iodine atom). The spectra were acquired with a recycle delay of 10 s. A 90° - 180° - 90° background suppression sequence was used to remove probe background and irradiation was centered at -130 ppm.[9]

The spectra shown in Supplementary Figure S10 were acquired on a Bruker 400 MHz spectrometer (376 MHz for ^{19}F NMR, with an AVANCE Neo console using a BBFO probe, and are reported in ppm. For ^{19}F NMR, a relaxation delay (D1) of 10 seconds was used, with a pulse angle of 90° to maximise SNR. 8,000 scans and 10,000 scans were collected over a spectral width of -300 to 100 ppm, and the Bruker inverse-gated ^1H decoupled pulse sequence (zgig) was used.

4.1. NMR analysis

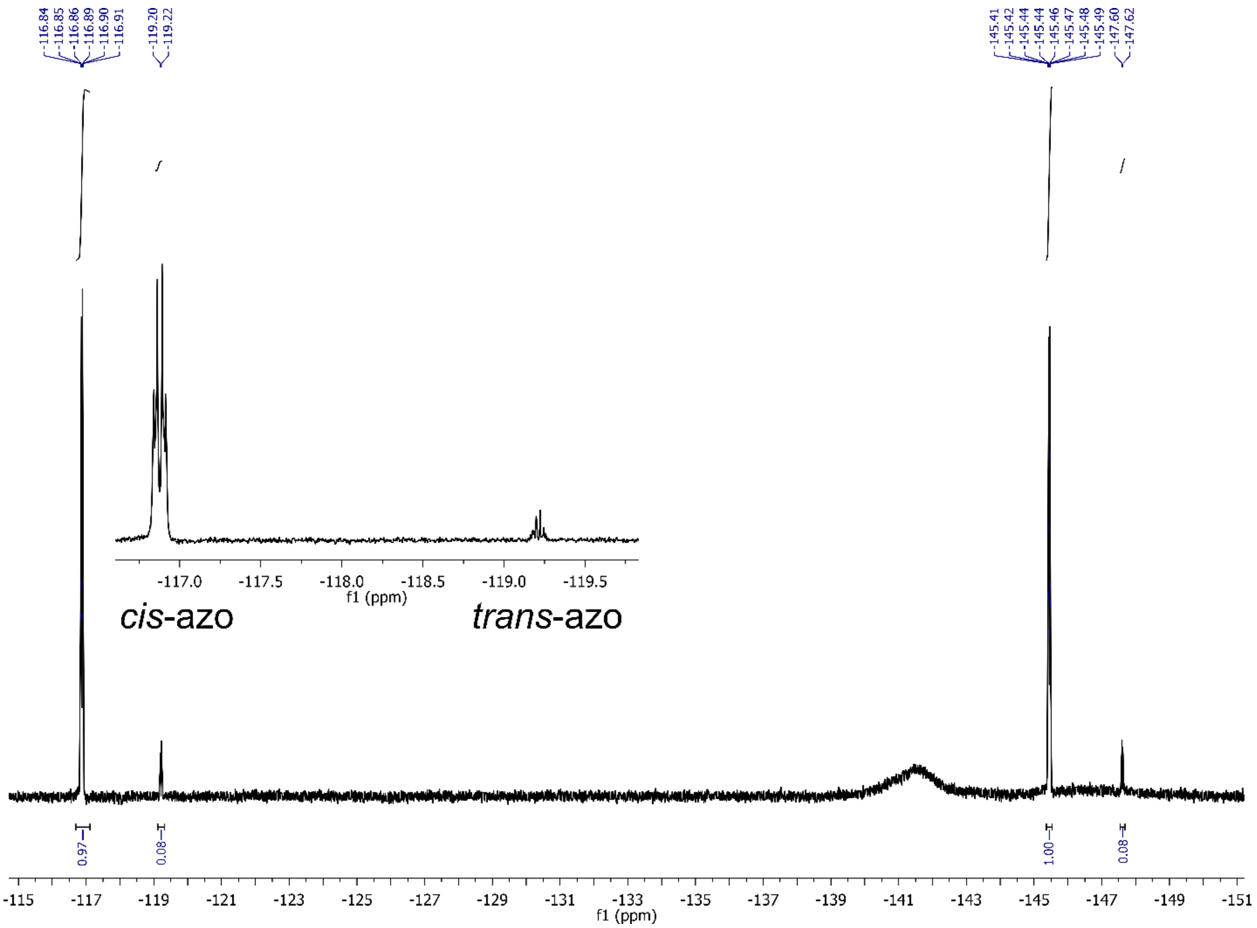

Supplementary Fig. 7. The ^{19}F NMR spectrum of a dissolved (*cis*-**azo**)(dioxane) single crystal after 20 min of irradiation by dispersed LED light.

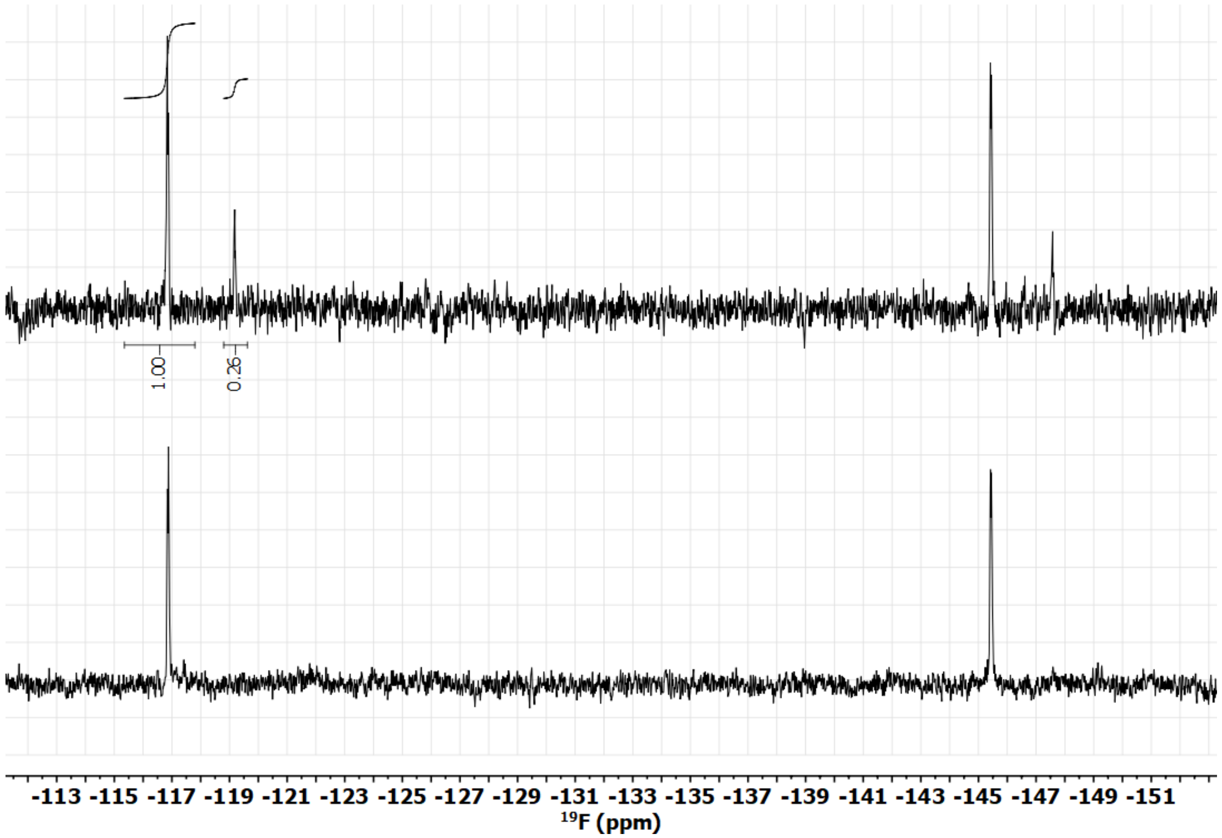

Supplementary Fig. 8. Comparison of ^{19}F NMR spectra measured for: (top) a dissolved (*cis*-**azo**)(dioxane) single crystal after approximately 1 minute irradiation using a 532 nm 150 mW laser and (bottom) a dissolved single crystal of (*cis*-**azo**)(dioxane) without any irradiation.

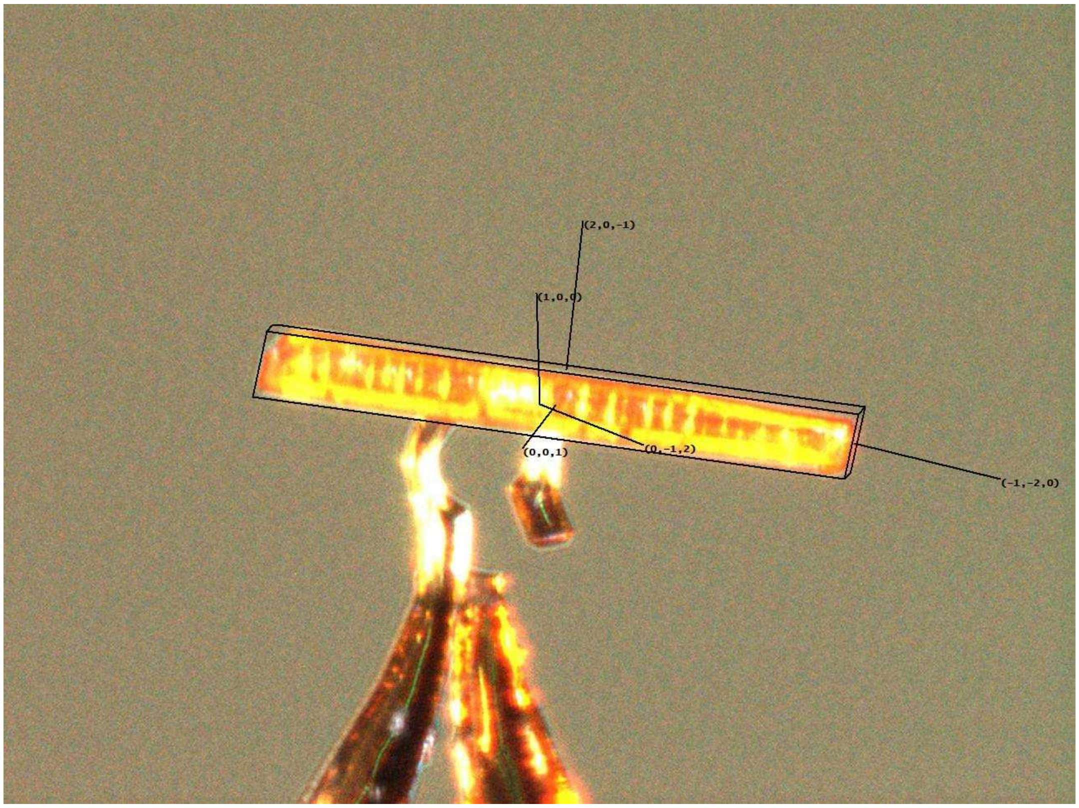

Supplementary Fig. 9. Crystal of (*cis*-**azo**)(dioxane) with the long-needle axis corresponding to the (001) lattice plane.

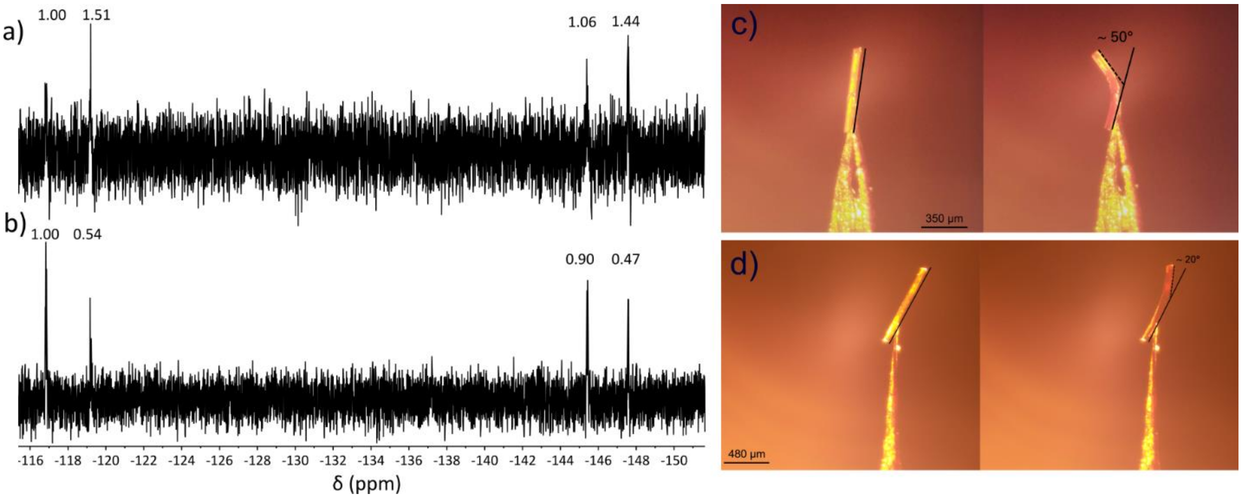

Supplementary Fig. 10. Comparison of ^{19}F NMR spectra for (*cis*-**azo**)(dioxane) single crystals after being bent to various degrees. Top image shows: a) the ^{19}F NMR spectrum; b) the single crystal before bending and c) the single crystal after bending to ca. 50°. Bottom image shows: a) the ^{19}F NMR spectrum; b) the single crystal before bending and c) the single crystal after bending to ca. 50°.

4.2. Raman spectroscopy:

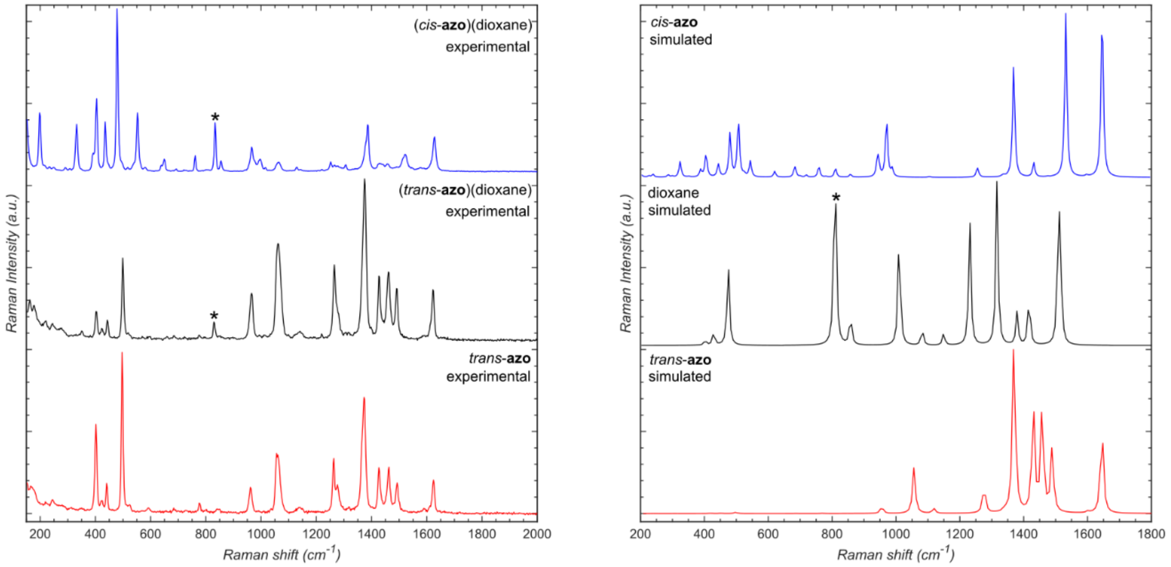

Supplementary Fig. 11. **(**right) Experimental Raman spectra of (*cis*-**azo**)(dioxane), (*trans*-azo)(dioxane) and *trans*-azo **II** acquired with a 785 nm probe, at 25 mW power, 2.5 s integration, over 10 accumulations. (left) Simulated gas-phase Raman spectra of dioxane, *cis*-**azo** and *trans*-**azo**. The asterisk (*) indicates the position of the dioxane *ν*(ring breathing) Raman band.

Supplementary Table 2. Assigned Raman shifts.

(*cis*-**azo**)(dioxane) experimental shifts (cm^{-1})	(*trans*-**azo**)(dioxane) experimental shifts (cm^{-1})	*trans*-**azo** experimental shifts (cm^{-1})	*cis*-**azo** simulated shifts (cm^{-1})	dioxane simulated shifts (cm^{-1})	*trans*-**azo** simulated shifts (cm^{-1})	assignment
336	N/A	N/A	324	N/A	N/A	*ν*(Aromatic)
N/A	350	N/A	N/A	N/A	N/A	
N/A	402	402	N/A	N/A	N/A	
410	N/A	N/A	405	N/A	N/A	*ν*(Aromatic)
N/A	423	423	N/A	N/A	413	*ν*(Aromatic)
437	443	440	443	N/A	444	*ν*(Aromatic)
501	499	497	506	N/A	491	*ν*(Aromatic), *ν*(N=N)
557	N/A	N/A	544	N/A	N/A	*ν*(Aromatic), *ν*(N=N)
N/A	778	776	N/A	N/A	N/A	
833	830	N/A	N/A	810	N/A	*ν*(ring breathing), dioxane
970	966	961	971	N/A	950	*ν*(Aromatic)
N/A	1061	1056	N/A	N/A	1050	*ν*(C−N)
N/A	1265	1263	N/A	N/A	1270	*ν*(C−N)

N/A	N/A	1276 Sh	N/A	N/A	N/A	
N/A	1375	1373	N/A	N/A	1364	*v*(N=N)
1368	N/A	N/A	1387	N/A	N/A	*v*(Aromatic)
N/A	1426	1426	N/A	N/A	1424	*v*(N=N)
N/A	1461	1463	N/A	N/A	1452	*v*(N=N)
N/A	1492	1492	N/A	N/A	1484	*v*(N=N), *v*(Aromatic)
1525	N/A	N/A	1532	N/A	N/A	*v*(N=N)
1634	1622	1624	1648	N/A	1640	*v*(Aromatic)

4.3. Scanning electron microscopy:

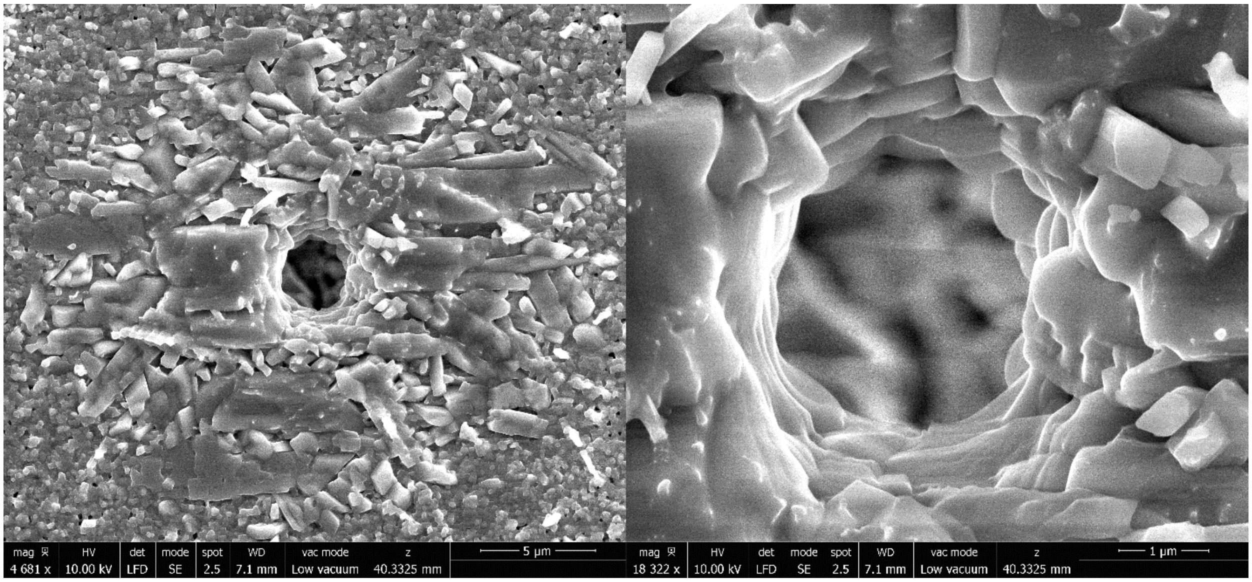

Supplementary Fig. 12. SEM images of (*cis*-**azo**)(dioxane) after carving showing precision hole formation, *via* a 532 nm 1 mW confocal laser system.

4.4. High speed camera studies:

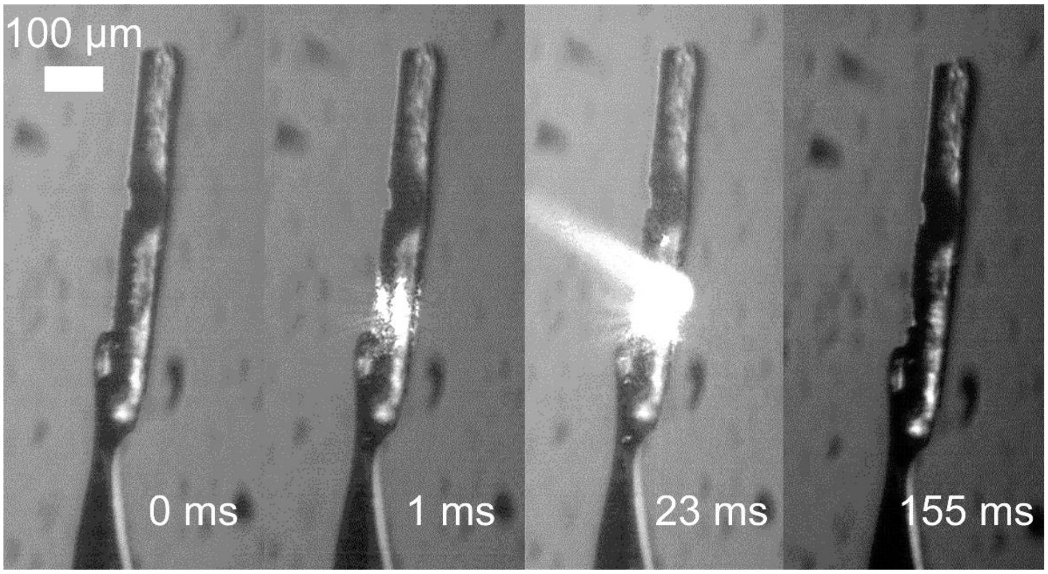

Supplementary Fig. 13. Images of a machining process of (*cis*-**azo**)(dioxane) captured *via* a high speed camera using a 532 nm 5 mW laser. See also Supplementary Video 5.

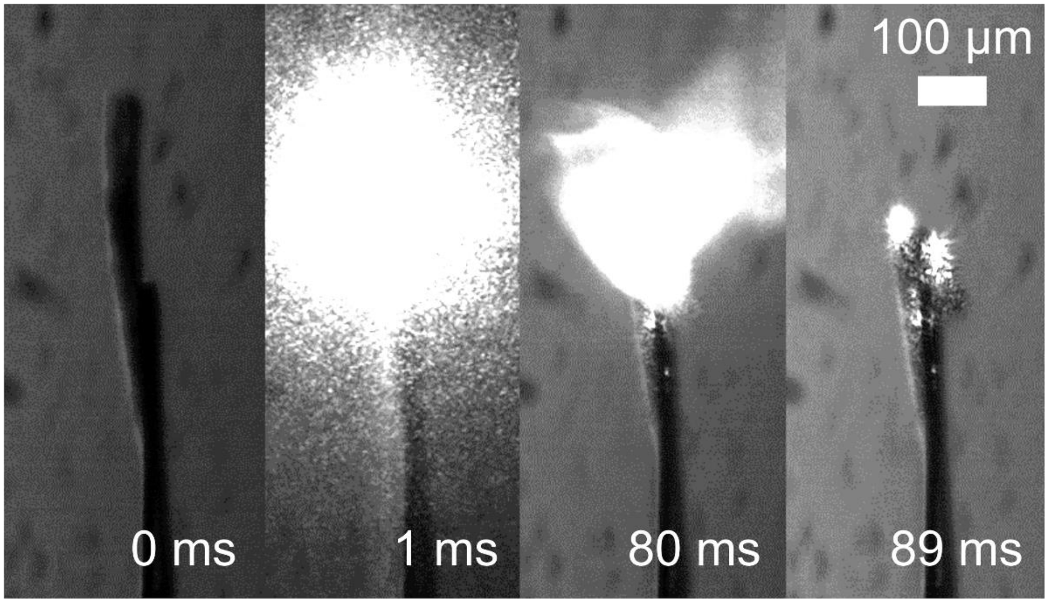

Supplementary Fig. 14. Images of a machining process of (*cis*-**azo**)(dioxane) captured *via* a high speed camera using a 532 nm 15 mW laser. See also Supplementary Video 3.

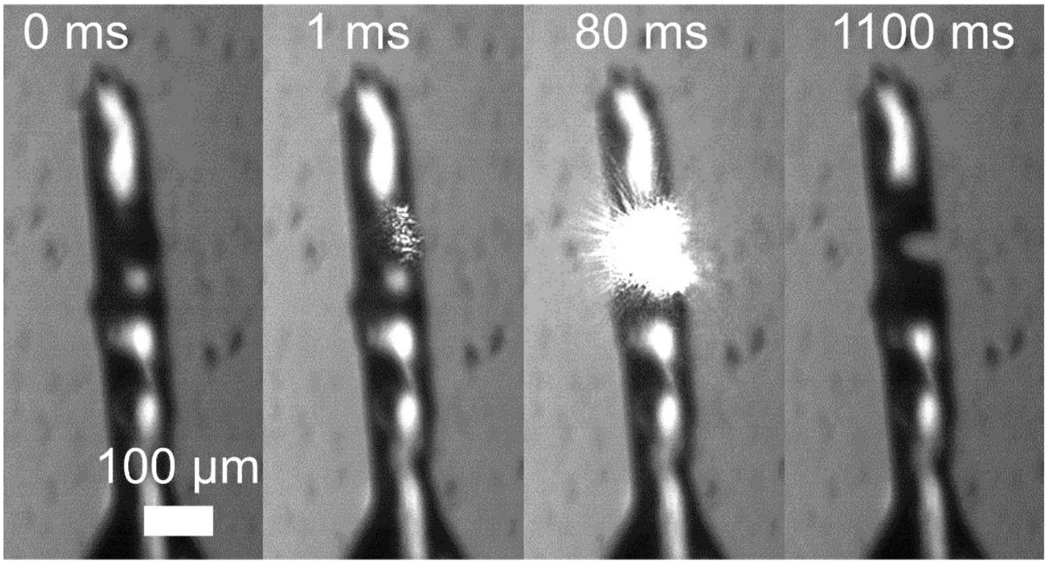

Supplementary Fig. 15. Images of a machining process of (*cis*-**azo**)(dioxane) captured *via* a high speed camera using a 532 nm 10 mW laser. See also Supplementary Video 4.

5. (*cis*-azo)(pyrazine) cocrystals:

5.1. NMR analysis:

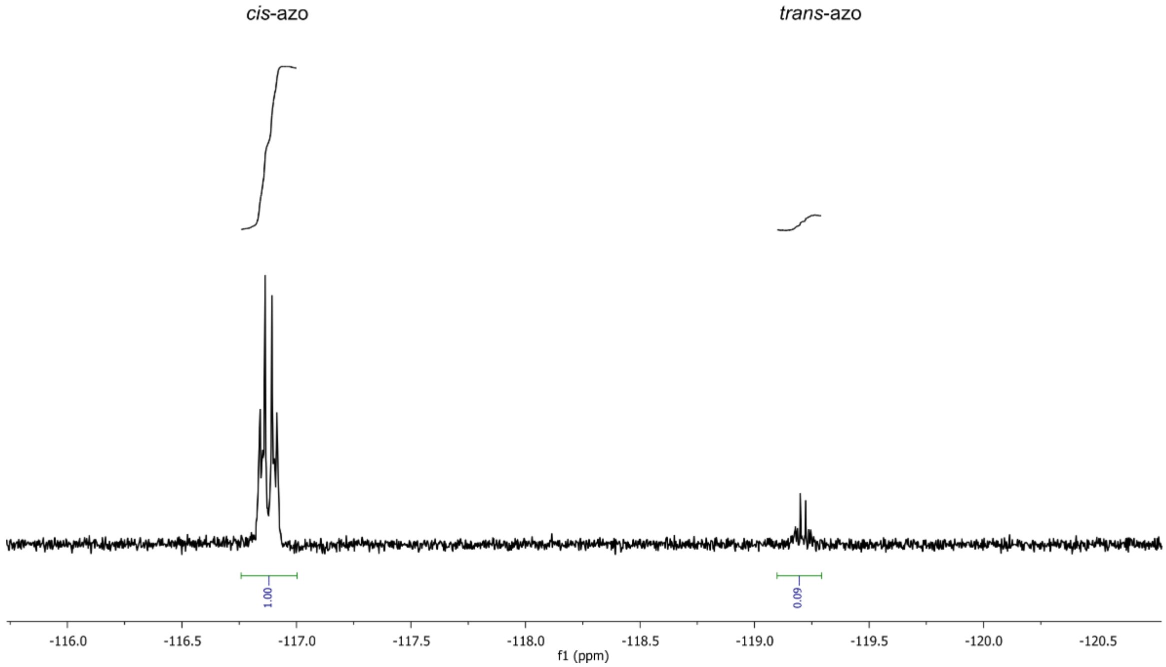

Supplementary Fig. 16. The ^{19}F NMR spectrum of a dissolved (*cis*-**azo**)(pyrazine) single crystal after 2 hours of irradiation.

5.2. Crystallographic studies of photo-carving of single crystals:

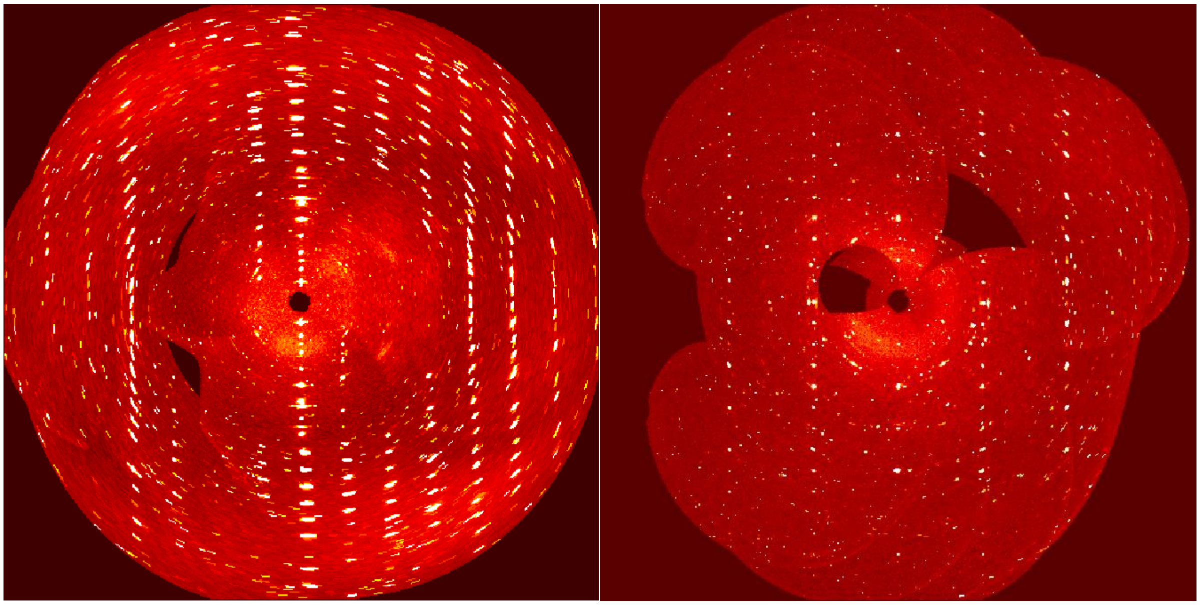

Supplementary Fig. 17. Precision images of (*cis*-**azo**)(pyrazine) showing the *0kl* layers: (left) before and after (right) photo-carving by a 532 nm laser at 20 mW power. A portion of the crystal was fully removed by laser light.

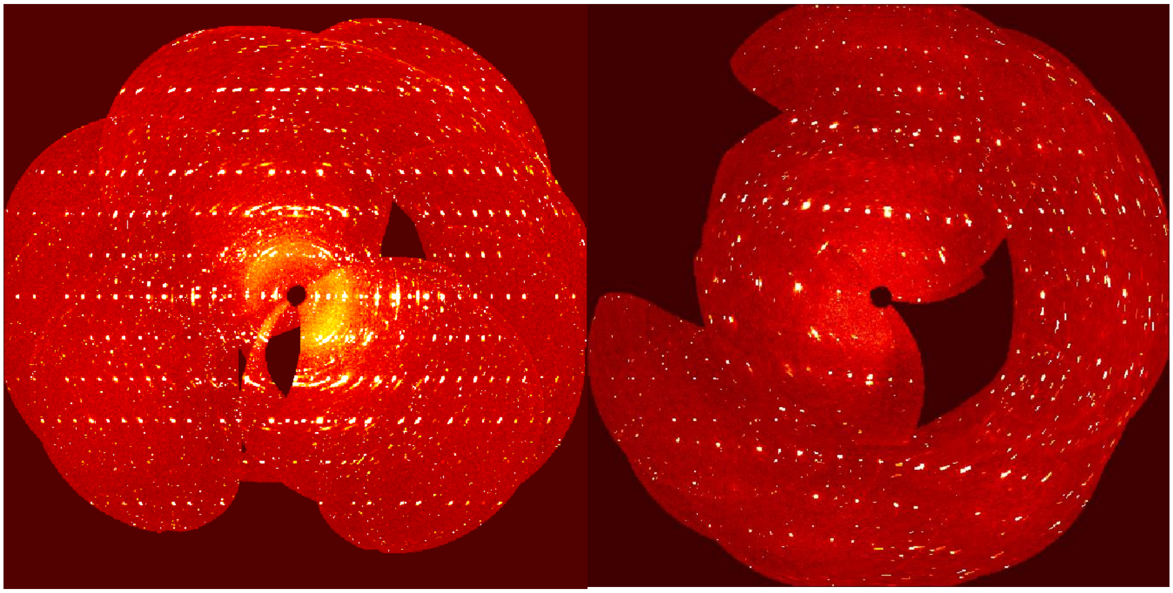

Supplementary Fig. 18. Precision images of (*cis*-**azo**)(pyrazine) showing the *hk0* layers: (left) before and after (right) photo-carving by a 532 nm laser at 20 mW power. A portion of the crystal was fully removed by laser light.

5.3. X-ray diffraction analysis:

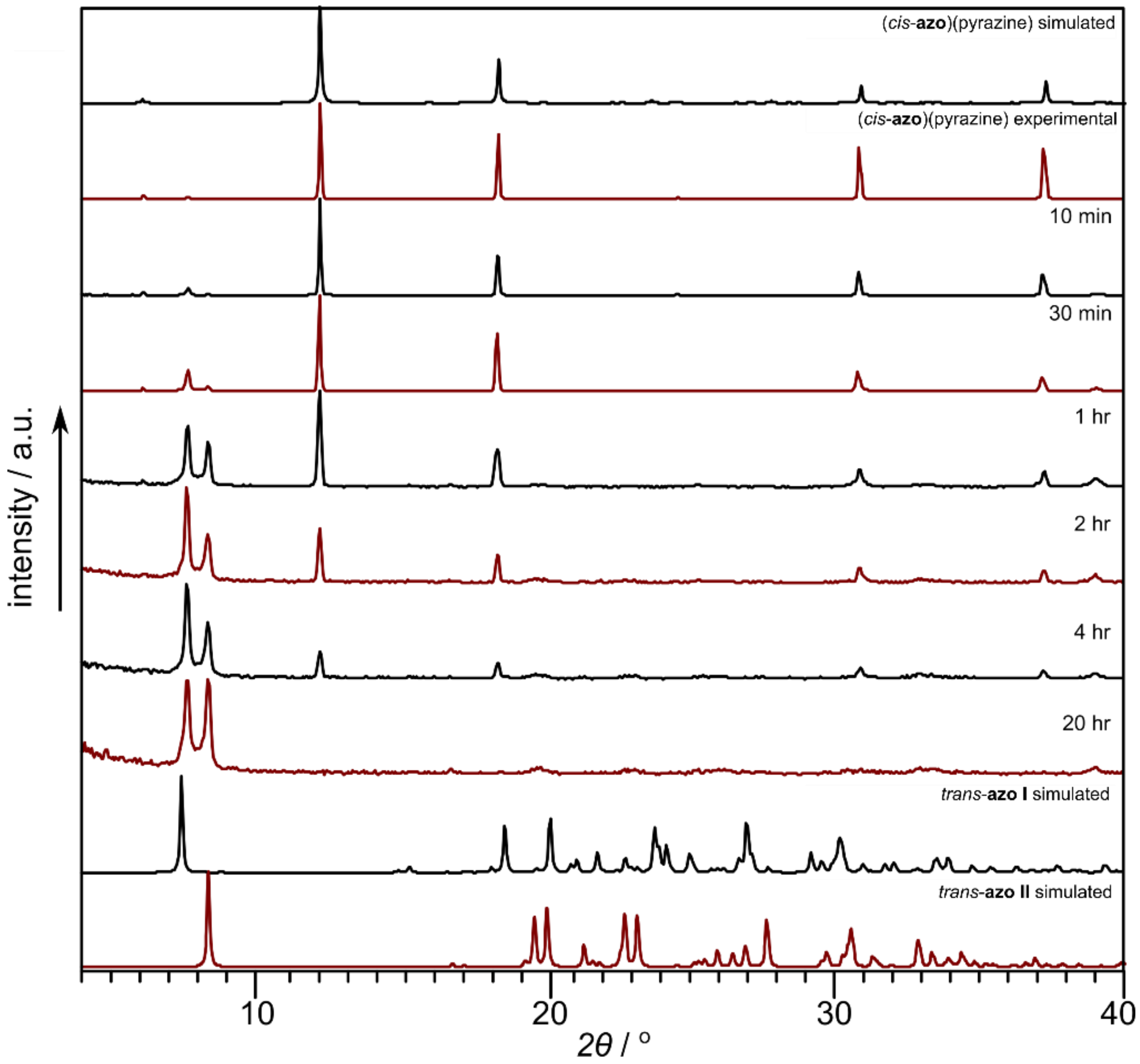

Supplementary Fig. 19. Time evolution of powder X-ray diffraction patterns of a bulk sample of (*cis*-**azo**)(pyrazine) under irradiation by a 532 nm 37 mW·cm^{-2} LED.

5.4. Scanning electron microscopy analysis:

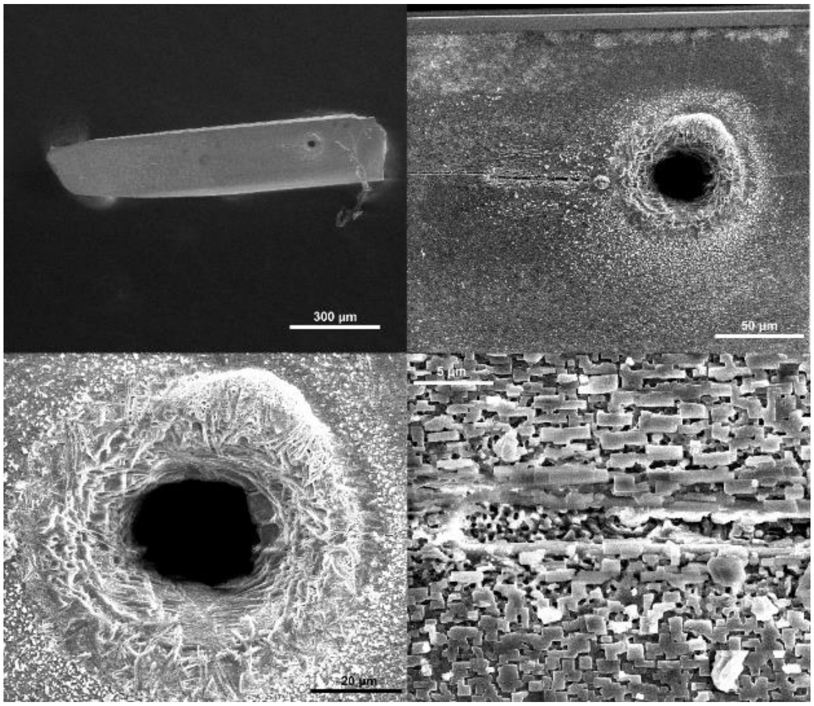

Supplementary Fig. 20. SEM images of (*cis*-**azo**)(pyrazine) after photo-induced carving by a 532 nm 15 mW confocal laser system (top right/bottom left), and photo-induced carving by a 532 nm 3 mW confocal laser system (bottom right).

5.5 Computational analysis of cocrystals:

Supplementary Table 3. Periodic density functional theory results of models discussed in this study.

**Model**	**Energy per unit cell /eV**	**Number of molecules in the cell**	**Energy per formula unit /eV**
(*trans*-**azo**)(dioxane)	-10963.46	2 (1 *trans*, 1 dioxane)	-10963.46
(*cis*-**azo**)(dioxane)	-21926.21	4 (2 *trans*, 2 dioxane)	-10963.11
(*trans*-**azo**)(pyrazine) (model reduced sym to $P2_1/n$ (parallel azos))	-21147.07	4 (2*trans*, 2 pyrazine)	-10573.54
(*cis*-**azo**)(pyrazine)	-21146.16	4 (2 *cis*, 2 pyrazine)	-10573.08
pyrazine (crystal)	-2496.19	2	-1248.10
pyrazine (g)	-1247.02	1	-1247.02
dioxane (g)	-1637.18	1	-1637.18
trans-**azo I**	-37301.68	4	-9325.42
trans-**azo II**	-18650.83	2	-9325.415
cis-**azo**	-18650.06	2	-9325.03

Supplementary Table 4. Formation energy calculations of various models discussed in this study.

Formation energies	Energy /eV	Energy /kJ/mol
(*cis*-***azo***) + pyrazine to (*cis*-***azo***)(pyrazine)	0.040685	3.92553291
(*trans*-***azo*** I) + pyrazine to (*trans*-***azo***)(pyrazine)	-0.02099	-2.02524114
(*trans*-***azo*** II) + pyrazine to (*trans*-***azo***)(pyrazine)	-0.028265	-2.72717679

6. Thermodynamic studies:

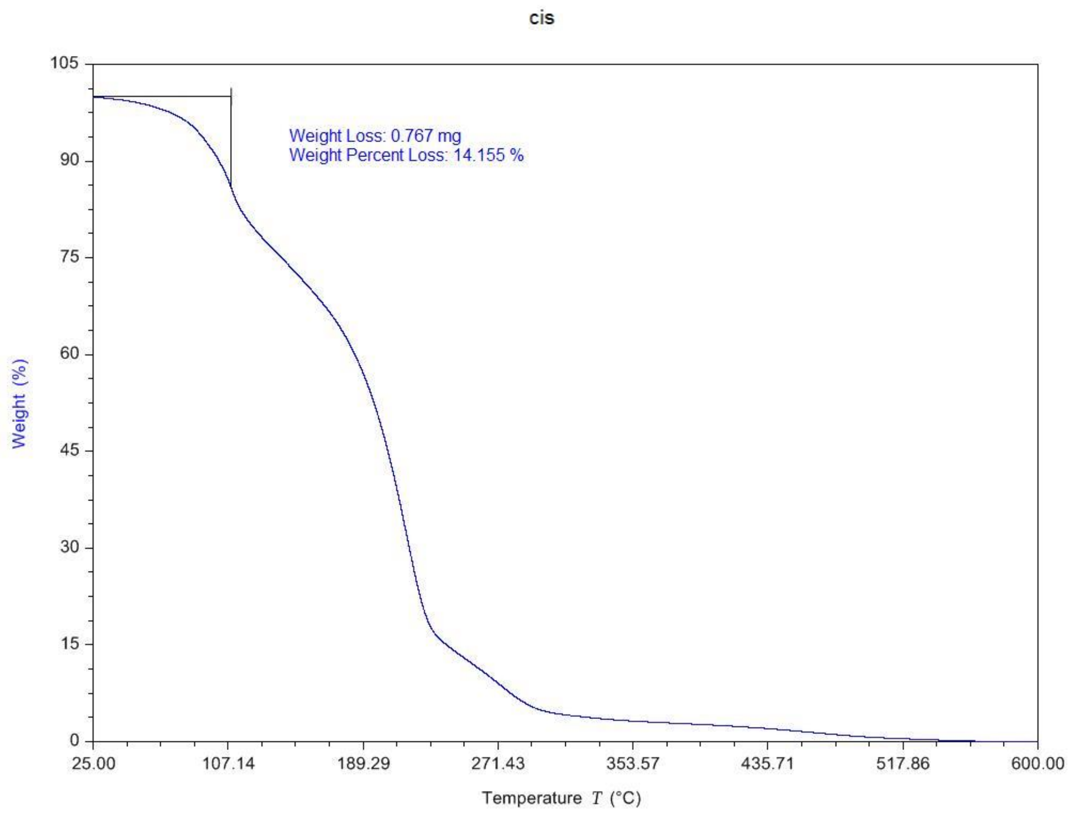

Supplementary Fig. 21. Thermogravimetric analysis of (*cis*-**azo**)(dioxane).

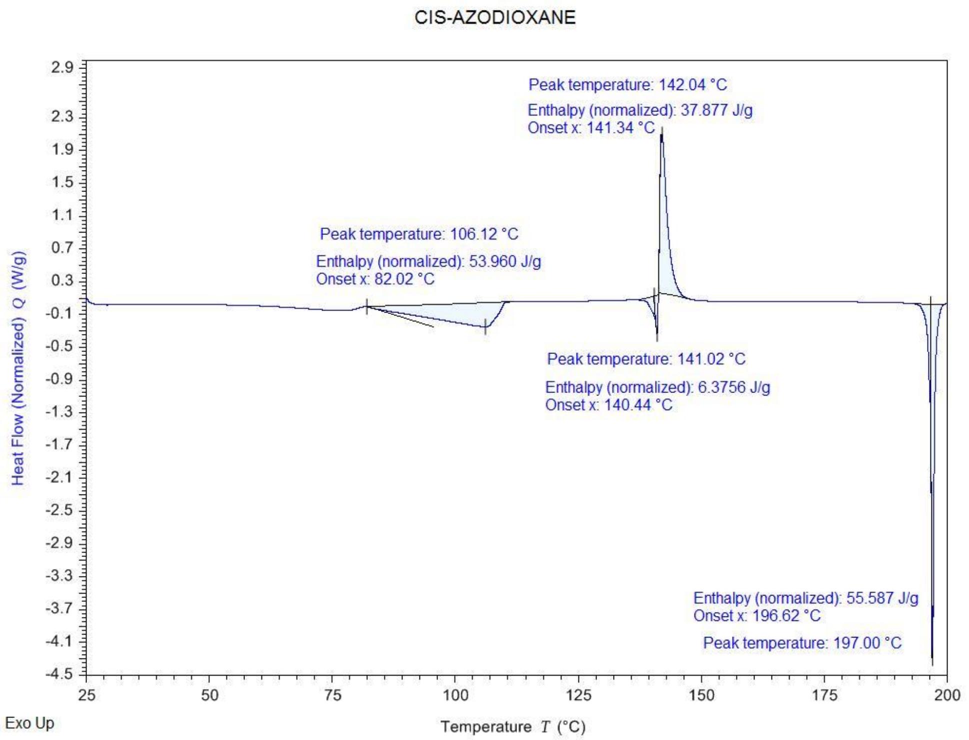

Supplementary Fig. 22. Differential scanning calorimetry analysis of (*cis*-**azo**)(dioxane).

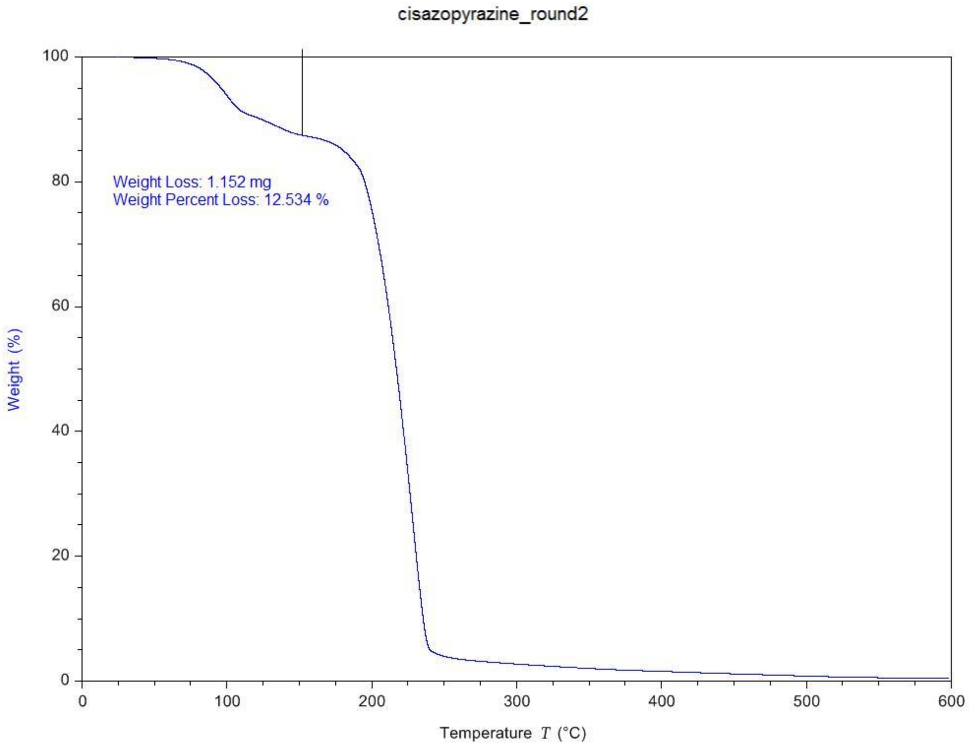

Supplementary Fig. 23. Thermogravimetric analysis of (*cis*-**azo**)(pyrazine).

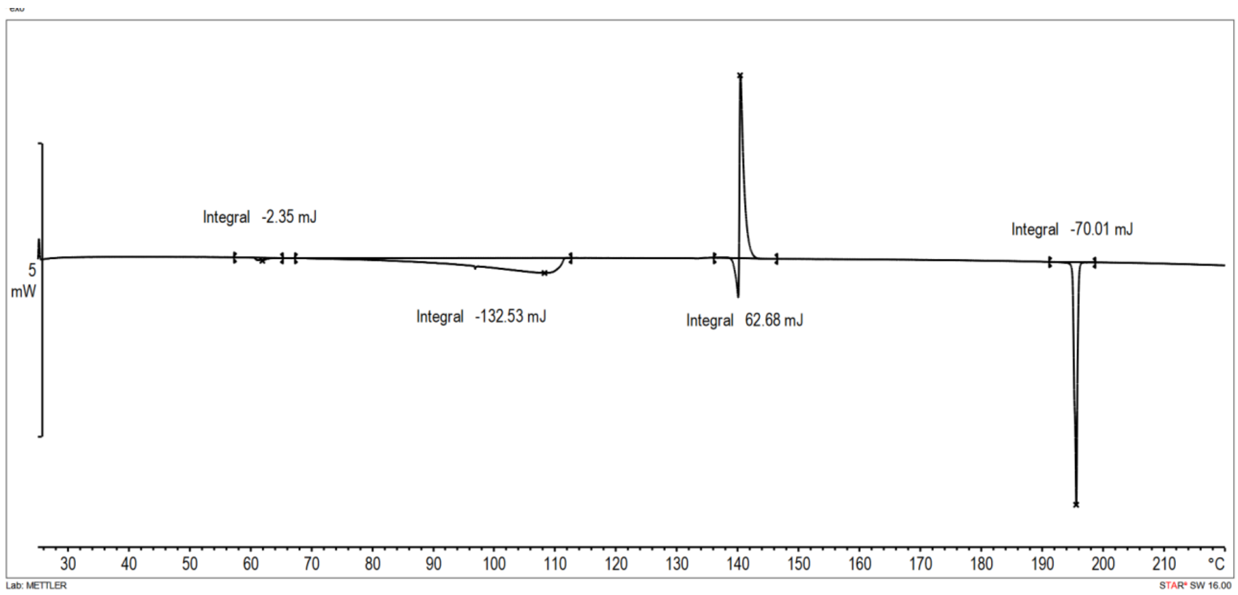

Supplementary Fig. 24. Differential scanning calorimetry analysis of (*cis*-**azo**)(pyrazine).

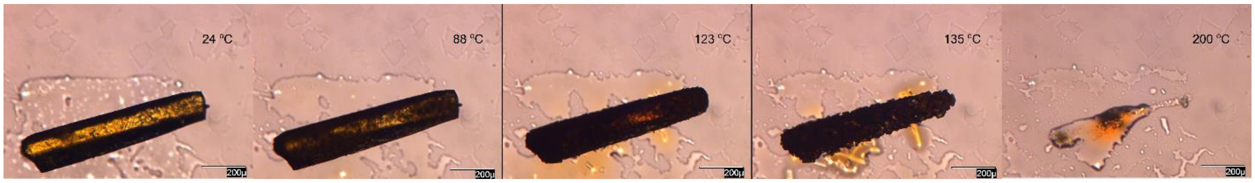

Supplementary Fig. 25. Hot-stage microscopy images of (*cis*-**azo**)(dioxane).

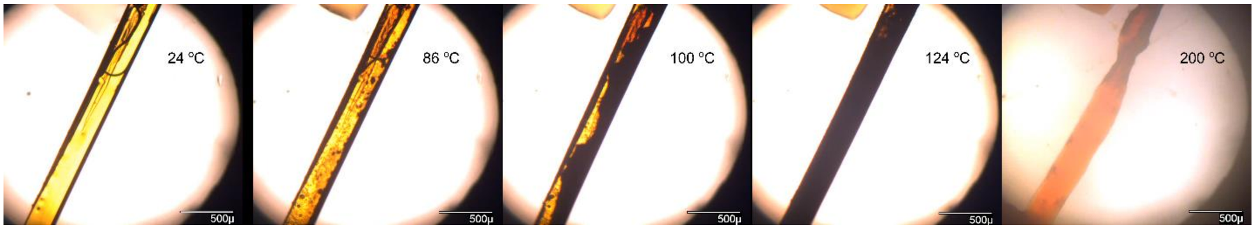

Supplementary Fig. 26. Hot-stage microscopy images of (*cis*-**azo**)(pyrazine).

7. Additional photo-carving images and movies of *cis*-azo cocrystals:

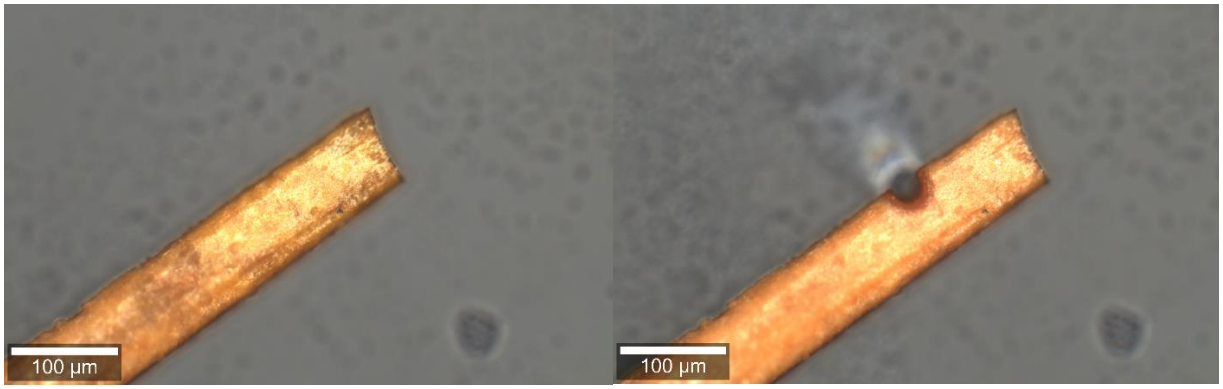

Supplementary Fig. 27. (*cis*-**azo**)(dioxane) before (left) and after (right) photo-irradiation using a confocal Raman 532 nm at 10 mW laser power for 1s duration.

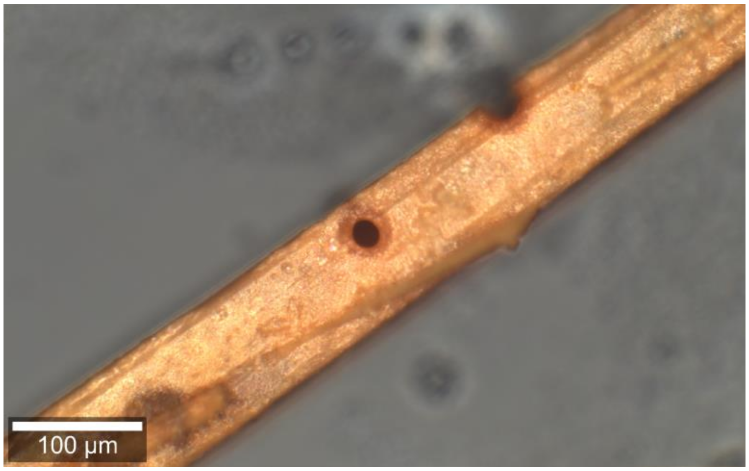

Supplementary Fig. 28. (*cis*-**azo**)(dioxane) after photo-irradiation using a confocal Raman 532 nm at 5 mW laser power for 1s duration.

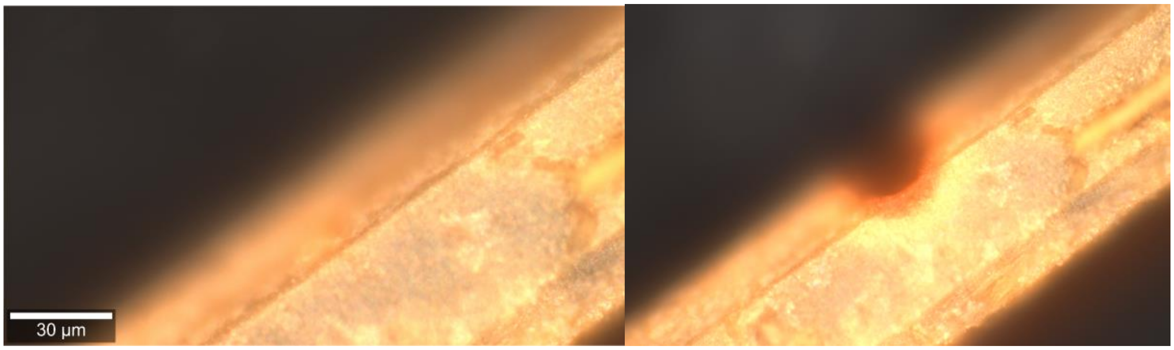

Supplementary Fig. 29. (*cis*-**azo**)(dioxane) after photo-irradiation using a confocal Raman 532 nm at 2.5 mW laser power for 1s duration.

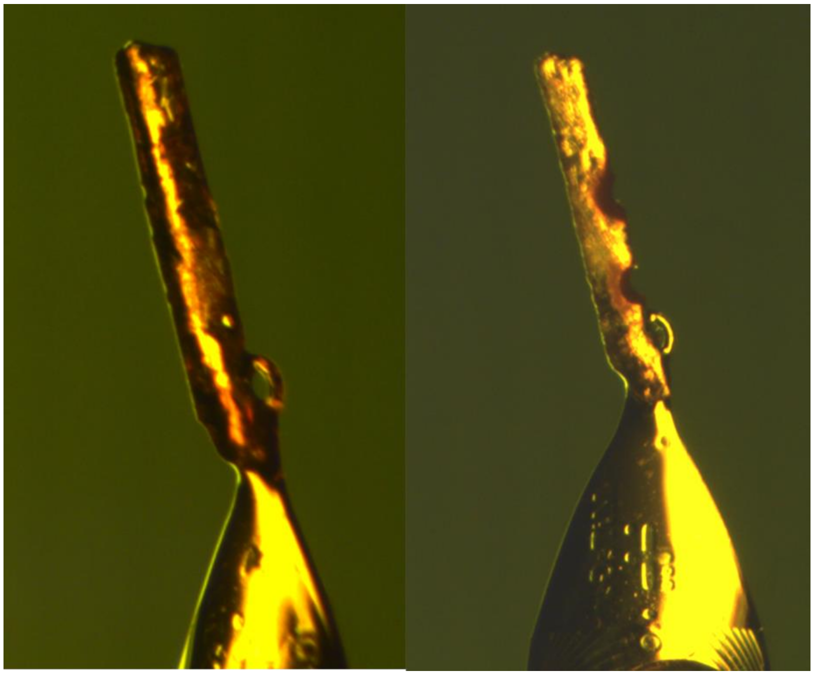

Supplementary Fig. 30. (*cis*-**azo**)(dioxane) before (left) and after (right) photo-irradiation using the laboratory laser system (SI Fig. 4) at 2.25 W cm^{-2} laser power. Red colour around the cuts indicates *cis*→*trans* isomerization.

7.1. Supplementary video and data file information:

Video 1. Photo-mechanical bending of (*cis*-**azo**)(dioxane) produced at 4× speed.

Video 2. Photo-carving of (*cis*-**azo**)(dioxane).

Video 3. High-speed camera video of (*cis*-**azo**)(dioxane) photo-carving 15 mW 532 nm irradiation.

Video 4. High-speed camera video of (*cis*-**azo**)(dioxane) photo-carving 10 mW 532 nm irradiation.

Video 5. High-speed camera video of (*cis*-**azo**)(dioxane) photo-carving 5 mW 532 nm irradiation.

Video 6. Photo-mechanical bending of (*cis*-**azo**)(pyrazine), video produced at 4x speed.

Video 7. Photo-carving of (*cis*-**azo**)(pyrazine).

8. References:

1. Bruker, APEX3, Bruker AXS Inc., Madison, Wisconsin, USA, 2012.

2. Krause, L., Herbst-Irmer, R., Sheldrick, G. M. & Stalke, D. Comparison of Silver and Molybdenum Microfocus X-ray Sources for Single-crystal Structure Determination. *J. Appl. Cryst.* **48**, 3–10 (2015).

3. Sheldrick, G. M. SHELXT - Integrated Space-Group and Crystal-Structure Determination. *Acta Cryst.* **A71**, 3–8 (2015).

4. Sheldrick, G. M. Crystal Structure Refinement with SHELXL. *Acta Cryst.* **C71**, 3–8 (2015).

5. Dolomanov, O. V., Bourhis, L. J., Gildea, R. J., Howard, J. A. K. & Puschmann, H. OLEX2: A Complete Structure Solution, Refinement and Analysis Program. *J. Appl. Cryst.* **42**, 339–341 (2009).

6. Farrugia, L. J. WinGX and ORTEP for Windows: an Update. *J. Appl. Cryst.* **45**, 849–854 (2012).

7. C. F. Macrae et al. Mercury 4.0: from Visualization to Analysis, Design and Prediction. *J. Appl. Cryst.*, **53**, 226–235 (2020).

8. Persistence of Vision Pty. Ltd., Persistence of Vision Raytracer, Persistence of Vision Pty. Ltd., Williamstown, Victoria, Australia, (2018).

9. Cory, D. G.; Ritchey, W. M. Suppression of signals from the probe in bloch decay spectra. *J. Magn. Reason.* **1969**, *80*, 128-132.
